# Uncovering traditional retail location competition with machine learning: The role of urban environments in light-asset and capital-intensive formats

**DOI:** 10.1371/journal.pone.0358597

**Published:** 2026-09-21

**Authors:** Yueyi Tan, Jusheng Song, Guangying Zhao, Yunxi Bai, Yan Li, Xuren Wei

**Affiliations:** 1 School of Architecture, Harbin Institute of Technology (Shenzhen), Shenzhen, China; 2 College of Architecture and Urban Planning, Tongji University, Shanghai, China; 3 Department of Architecture, College of Design and Engineering, National University of Singapore, Singapore; Beijing Technology and Business University, CHINA

## Abstract

Understanding spatial competition between retail formats is vital for sustaining urban commercial vitality. In China’s rapidly evolving urban economy, the contrast between light‑asset and capital‑intensive formats within traditional retail has been increasingly shaped by urban redevelopment and changing consumer behavior. However, prior studies largely confine themselves to linear assumptions, neglecting nonlinear dynamics and complex interdependencies. They also lack systematic comparisons between light-asset and capital-intensive formats, especially when attempting to integrate objective urban features with perceptual dimensions. This study investigates how objective urban environment indicators and human perception jointly shape the spatial density of two traditional retail formats, light‑asset (convenience stores) and capital‑intensive (large-scale supermarkets and shopping malls), in Shenzhen, China. Using multi‑source geospatial data and an integrated framework combining XGBoost, SHAP, Partial Dependence Plots (PDP), Interpretive Structural Modeling (ISM), and Bayesian Networks (BN), we quantify nonlinear effects, interaction patterns, and probabilistic association pathways. Results reveal distinct locational logics: light‑asset retail thrives through widespread penetration, responding strongly to competition intensity, service‑function proximity, and perceptual synergies; capital‑intensive retail concentrates in high‑yield hubs, driven by facility networks, accessibility, and high‑quality aesthetic environments above defined thresholds. Human perception emerges as a core determinant, accounting for up to 28.95% of explanatory importance, often exerting threshold‑dependent effects. The ISM–BN analysis uncovers multi‑entry vs. sequential pathways to spatial clustering across formats. Findings advance retail location theory by linking built‑environment metrics with street-level perceptual attributes, offering actionable guidance for urban planners to tailor format‑specific, perception‑oriented, and threshold‑sensitive strategies for sustainable retail ecosystems.

## 1. Introduction

The retail sector is a cornerstone of urban economies, profoundly influencing city structure, consumer behavior, and socio-economic development [1,2]. Traditional retail, an important type of retail encompassing diverse offline formats, offers a wide range of goods and services and form the backbone of urban everyday life [3]. At the heart of traditional retail success lies the strategic choice of store location, as location decisions determine not only the accessibility of goods and services but also the vibrancy and sustainability of urban commercial environments [4]. With the ongoing development of traditional retail, variations in scale and capital intensity have become increasingly pronounced, leading to differentiated patterns of location strategy. Understanding the dynamics of location choice among different formats within traditional retail, particularly those varying in scale and capital intensity, is crucial for decoding contemporary urban retail competition, informing urban planning, and fostering competitive yet inclusive commercial landscapes.

This study focuses on traditional retail that provides essential goods and services, such as food, clothing, daily necessities, and convenience items, which plays a critical role in sustaining urban residents’ well-being and has long been a focus of interdisciplinary research [5,6]. In analyzing retail operations, differences in capital intensity are often used to classify business models [6–10]. Drawing on established retail geography and retail operation studies [7], this study stratifies offline traditional retail into two mutually exclusive categories anchored to asset intensity as the core classification criterion, based on four core dimensions: capital input scale, fixed asset investment, site operation constraints, and spatial expansion logic. The light-asset format is represented by convenience stores: characterized by small store footprints, low upfront rental and construction costs, flexible site screening criteria, low market entry thresholds, and decentralized, neighborhood-oriented spatial penetration. In the Chinese urban context, nearly all mainstream convenience store chains have integrated online ordering, instant delivery and O2O service modules. However, digital operations serve as supplementary revenue channels rather than reshaping their fundamental physical-site-dependent competition logic. This study focuses on the spatial density and locational competition of offline store entities, where pedestrian flow, street environmental perception and surrounding physical facilities remain dominant decision-making factors for retailers. Thus, the asset-intensity-based classification framework retains analytical validity despite hybrid online-offline business models. The capital-intensive format aggregates large-scale chain supermarkets and integrated shopping malls. While these two sub-types diverge in tenant mix, consumer trip purposes and service coverage radius, they share four consistent asset-driven locational attributes: (1) substantial fixed capital investment in physical construction, decoration and long-term land leasing; (2) rigid site constraints requiring large contiguous land parcels; (3) reliance on large, stable catchment populations to amortize high fixed costs; (4) clustered development within high-value urban commercial hubs rather than scattered neighborhood deployment. For this reason, we group them as a unified capital-intensive category for core cross-format comparative analysis centered on asset intensity. These formats differ markedly in resource commitment, operational flexibility, and market positioning. While long coexisting in cities, recent trends in capital concentration and urban redevelopment have intensified spatial competition, with capital‑intensive formats securing prime urban locations and leveraging advantages in product quality and assortment, often constraining light‑asset operators [4,11]. Nevertheless, light‑asset retail contributes to grassroots entrepreneurship, employment generation, and accessible consumption, thereby supporting urban resilience and inclusiveness [12–14]. A nuanced understanding of their respective location strategies offers critical insight into how urban environment privileges certain retail formats over others, and into the consequences this holds for equitable access, community vitality, and the everyday quality of urban life.

Prior research has identified several key factors influencing traditional retail location choices. Demographic characteristics, such as population density, income level, education, and age structure, are commonly found to affect site attractiveness and market demand [3,15–17]. Accessibility, including proximity to transport links, road networks, and public transit, also plays a significant role [11]. Socioeconomic factors like rental cost, local economic activity, and competition intensity often shape retailer’ locational decisions and performance [3,6]. Additionally, the density and number of surrounding facilities, such as schools, companies, and residences, are relevant [11,18].

Despite these advances, several important gaps remain in the literature. First, prior research has rarely conducted a systematic comparison of light-asset and capital-intensive formats within traditional retail in terms of their location choices, nor examined how urban environmental factors differentially influence these choices [6,19,20]. Second, previous studies on retail location choice have largely ignored the influence of human perception [11], encompassing both visual perception and emotional perception, which cannot be fully captured by conventional urban environment metrics. Third, most existing research remains confined to examining straightforward correlations, offering limited insight into complex interrelationships, layered dependencies, and probabilistic association pathways that shape retail spatial patterns [3,11]. Taking convenience stores, and large‑scale supermarkets and shopping malls as representatives of light‑asset and capital‑intensive forms of traditional retail in Shenzhen, this study systematically investigates how both objective urban environmental characteristics and human perception shape the spatial density of traditional retail. By integrating XGBoost, SHAP, PDP, ISM, and BN into a multi-layered analytical framework, the research not only reveals nonlinear relationships but also uncovers complex interactions, hierarchical dependencies, and probabilistic association pathways underlying retail location choices of light-asset and capital-intensive models.

The remainder of this paper is structured as follows: Section 2 reviews relevant literature on retail location and its determinants; Section 3 describes the research methodology; Section 4 presents the results; Section 5 discusses the contributions, policy implications, research limitations, and future research trends; Section 6 concludes with a summary of key results.

## 2. Literature review

### 2.1. Retail location

The selection of retail store locations has long been recognized as a critical determinant of retail success. Classical spatial theories laid the foundation for understanding retail distribution and consumer attraction. Central Place Theory [21] and Reilly’s Law of Retail Gravitation [22] emphasized the roles of accessibility, store attractiveness, and spatial equilibrium in shaping retail patterns. Building on these ideas, Huff’s probabilistic trade-area model further highlighted consumer catchment and probabilistic store choice [23], while subsequent analog models [24], spatial interaction models [25], and econometric approaches [26] became widely adopted tools for predicting store performance and optimal siting. Together, these approaches established a robust theoretical basis for explaining how distance, market size, and surrounding competition influence retail location outcomes.

Retail location research has since evolved from classical spatial explanations toward increasingly data-driven and behaviorally nuanced approaches. Later studies extended the focus beyond distance and market potential, showing that store performance also depends on format differentiation, competitive structure, and local urban context [10,27]. In recent years, the retail landscape has diversified, marked by the proliferation of e-commerce and the rapid rise of “new retail” or online-to-offline (O2O) formats [11]. These developments have fundamentally influenced the criteria and mechanisms underlying retail location choice. Rather than diminishing the importance of physical location, the integration of digital channels, advanced logistics, and data-driven consumer insights has introduced new layers of complexity to location strategy [28,29]. This shift does not only affect new entrants; it also pressures established, traditional retail to adapt and refine their business models and location strategies. At the same time, it is worth noting that traditional retail itself is not a static category. With socio-economic transformation, demographic shifts, and evolving consumer preferences, the internal composition and operational focus of traditional retail have undergone notable changes [4,30]. Store formats have diversified, operational scales have become more polarized, and location strategies are increasingly differentiated according to capital investment and market positioning [6,16]. Existing studies have begun to reveal substantial heterogeneity across retail formats—for example, between specialty grocers and traditional supermarkets, or between new retail and conventional offline retail—suggesting that location logic varies systematically with business model, capital intensity, and target market [3,6,7]. For instance, research comparing Starbucks and Luckin Coffee has demonstrated that new retail brands adopting O2O models exhibit unique sensitivities to demographic, socioeconomic, and competitive factors compared to more established traditional retail [3,11]. In parallel, there is growing attention to the heterogeneity within traditional retail itself, with studies contrasting the location strategies of specialty grocers and traditional supermarkets in Western contexts [6]. These works reveal that even within brick-and-mortar retail, different subtypes, distinguished by product range, target demographics, and service models, respond differently to their urban and competitive environments.

However, despite these advances, there remains a significant gap in the literature regarding the spatial strategies of traditional retail differentiated by their operational scale and asset intensity. This is a critical omission, as the scale and asset structure of retail formats fundamentally shape their location logic, resilience to market changes, and roles in urban spatial structure. Consequently, it is necessary to systematically compare the location strategies of different traditional retail formats, particularly light -asset and capital‑intensive types.

### 2.2. Determinants of retail location in the urban context

A comprehensive array of objective factors has been shown to shape retail location choice. Demographic variables such as population density, income, education, household size, and age structure are consistent predictors of site attractiveness and sales performance [3,15–17]. Socioeconomic determinants, including rental costs, land prices, and economic activity, are particularly salient for capital-intensive retail formats [6,31]. Accessibility remains a foundational principle, with successful sites often characterized by proximity to transport nodes, arterial roads, and public transit [11]. The density and number of surrounding facilities, such as other retail outlets, offices, schools, and health amenities, have gained increased attention as markers of the vibrancy and drawing power of a locality [3,11]. Besides, recent research underscores the competitive environment as a critical factor.

However, beyond these objective environmental determinants, human perceptions of the environment, including factors such as perceived safety, beauty, and wealth, are increasingly recognized as influential in shaping retail location success [11]. Early urban design research demonstrated that perceived environmental quality is strongly associated with walkability and place experience [32]. With the development of street-view imagery and computer vision, urban perception can now be quantified at scale, including perceived safety, visual quality, and emotional impressions of streetscapes [33–35]. In the Chinese context, related studies have also begun to use large-scale street-view datasets to measure urban greenery and visual quality, confirming the value of human-scale environmental indicators in high-density cities [36,37]. While such subjective factors have been extensively studied in consumer behavior and environmental psychology [32,38], their explicit integration into traditional retail location models remains limited. Most existing empirical work continues to focus on quantifiable, external variables, overlooking the ways in which individual and collective perceptions mediate the relationship between urban context and retail performance. This omission is notable given recent evidence that environmental cognition and behavioral preferences can significantly modify both consumer flows and the effectiveness of retail siting [11].

Methodologically, the vast majority of classic literature relies on linear models, which may oversimplify the often complex and nonlinear effects of environmental determinants. The recent adoption of machine learning techniques, such as XGBoost, has enabled researchers to capture nonlinear patterns [3]. Recent advances in explainable artificial intelligence for tree-based models have further made it possible to move from predictive performance to interpretable understanding of feature contributions and interactions [39]. Integrated analytical pipelines combining XGBoost, SHAP, PDP and Bayesian Networks have proven effective for evaluating retail suitability while incorporating streetscape perceptual metrics, offering a solid methodological foundation for unpacking retail location mechanisms [40]. Related geospatial analytics focusing on retail density thresholds further highlight the value of bootstrap-based uncertainty testing for reliable identification of tipping points across multi-source spatial datasets [41]. In addition, studies focusing on commercial data analytics have demonstrated the great potential of large language models for mining heterogeneous commercial text information [42], which delivers new inspiration for enriching multi-dimensional input features in grid-scale retail spatial analysis. The integration of multi-source geospatial data, including POIs, mobile signaling, and socioeconomic statistics, further enhances the explanatory power of location choice models, particularly in new retail scenarios characterized by high consumer mobility and behavioral heterogeneity.

Despite these advances, several important gaps remain in the existing literature. First, although prior studies have compared retail subtypes such as new versus traditional retail or specialty grocers versus conventional supermarkets, few have systematically examined heterogeneity within traditional retail through the lens of asset intensity and operational scale. As a result, the distinct locational logics of light-asset and capital-intensive formats remain insufficiently understood. Second, while recent studies have begun to quantify human perception using street-view imagery and computer vision, such perceptual dimensions are still rarely integrated with objective urban-environment indicators in traditional retail location models. Third, current machine-learning applications in retail geography have mainly focused on prediction, variable importance ranking, or the identification of nonlinear effects, but have seldom provided an integrated framework capable of jointly revealing interaction effects, hierarchical dependencies, and probabilistic structural pathways. Fourth, much of the emerging evidence on perception-aware and data-driven retail location analysis is derived from new retail scenarios or non-Chinese contexts, leaving a relative lack of empirical evidence from high-density Chinese cities where intense competition, rapid mobility, and mixed urban functions may reshape retail locational mechanisms. Addressing these gaps, this study develops an integrated framework combining XGBoost, SHAP, PDP, ISM, and BN to compare light-asset and capital-intensive traditional retail in Shenzhen, and to examine how objective urban conditions and human perception jointly shape their spatial density patterns.

## 3. Data and methodology

### 3.1. Analytical framework and study area

**Fig 1:**
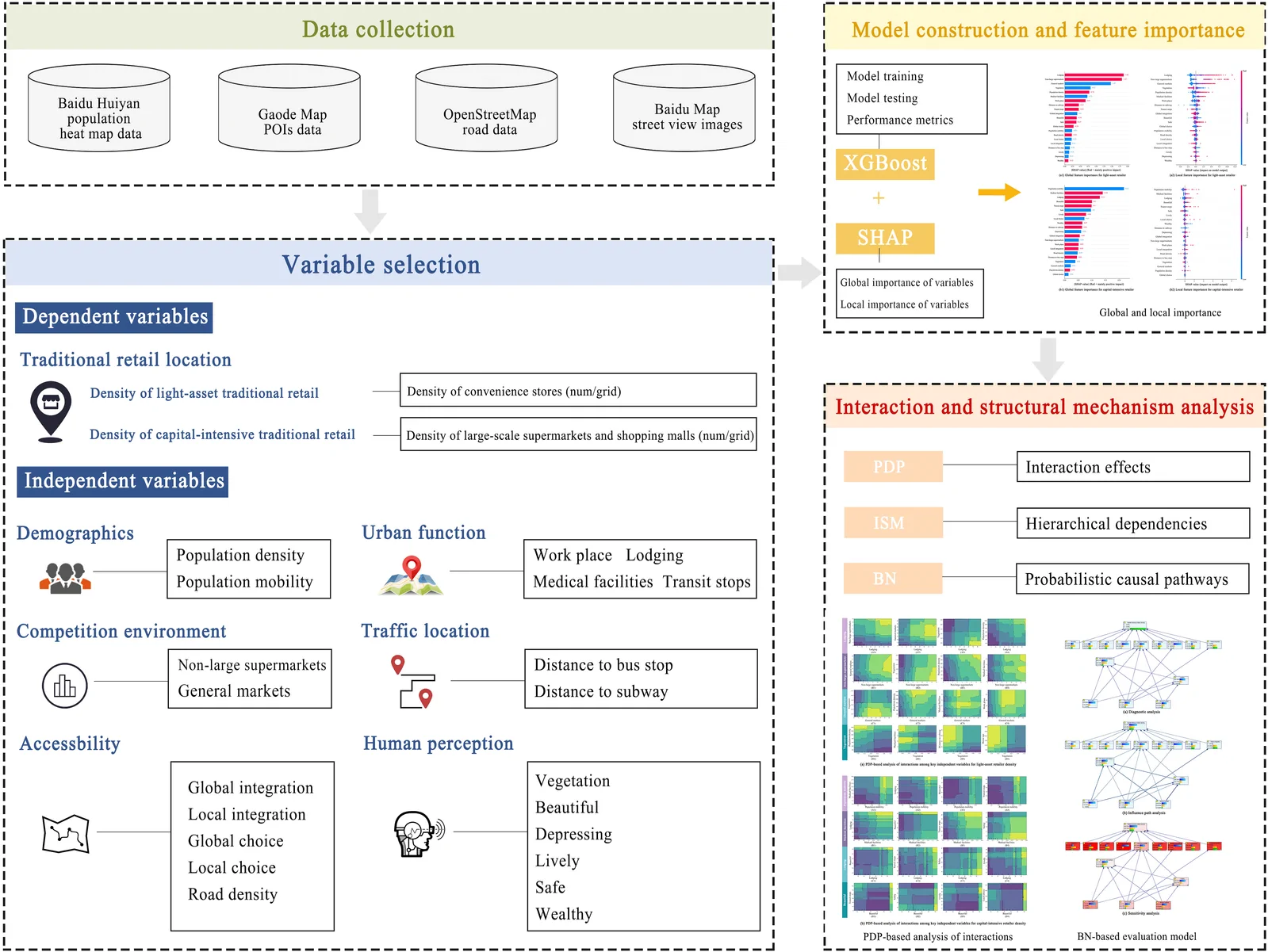
The analytical framework.

The analytical framework for this study is illustrated in Fig 1. It combines multi-source urban data collection, systematic variable selection, advanced machine learning modeling, and structural mechanism analysis to reveal the spatial logic underlying the location competition between light-asset and capital-intensive traditional retail formats.

**Fig 2:**
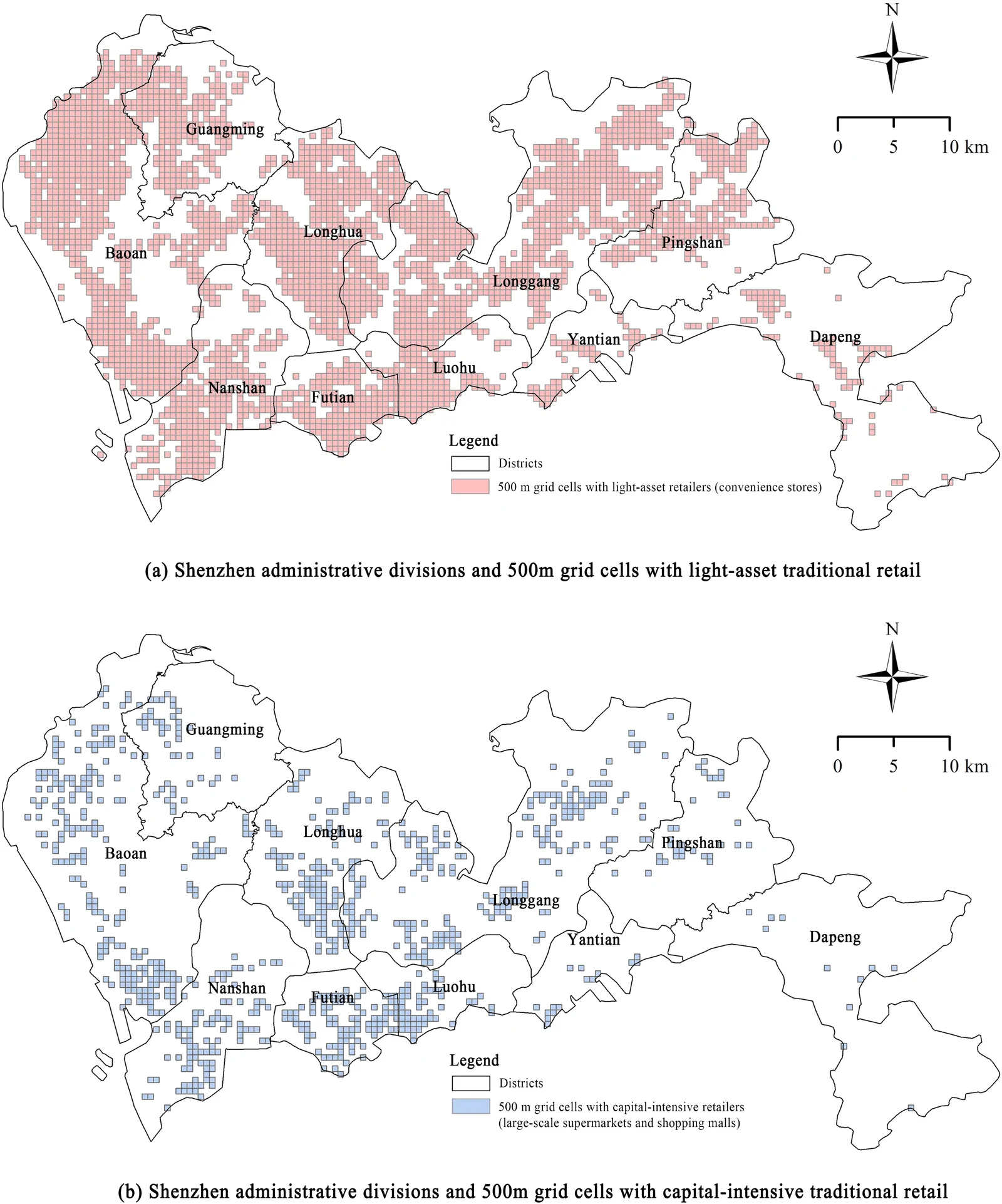
The study area. (a) Light-asset traditional retail: 500m grid Cells in Shenzhen. (b) Capital-intensive traditional retail: 500m grid cells in Shenzhen. Base map data from OpenStreetMap, available under the Open Database License (ODbL).

The empirical analysis is conducted in Shenzhen, a leading metropolis in southern China renowned for its rapid urbanization, diverse retail ecosystem, and pronounced spatial heterogeneity. Shenzhen represents an ideal case for examining retail location strategies due to its high population density, dynamic socioeconomic environment, and the coexistence of both light-asset and capital-intensive traditional retail. For spatial granularity, the study area is partitioned into uniform grid units. To mitigate potential bias caused by the modifiable areal unit problem, we implemented a spatial resolution sensitivity test with three uniform grid specifications (300 m, 500 m, and 800 m). We re-ran the full XGBoost-SHAP-PDP-ISM-BN workflow for each grid size and cross-compared variable importance rankings, critical threshold values and hierarchical association structures. The results show highly consistent spatial patterns, feature importance hierarchies and critical threshold intervals across the three grid resolutions. Quantitatively, pairwise Spearman’s rank correlation coefficients of SHAP variable importance rankings between different grid sizes all exceed 0.99 for both retail formats; detailed comparative variable importance tables, Spearman correlation values and bootstrapped threshold estimates with 95% confidence intervals are provided in S1–S6 Tables of the Supporting Information, verifying the robustness of core findings to spatial unit granularity. The 500-meter grid was ultimately selected as the primary analytical unit because it aligns with the typical walking distance for daily retail consumption in Chinese cities and balances sample volume with fine-grained spatial detail. This resolution captures subtle variations in the urban environment well while maintaining adequate statistical reliability in the retailer distribution data, consistent with widely adopted spatial analysis standards in existing urban studies [43]. In the baseline analysis, grid cells lacking either light-asset or capital-intensive traditional retail POIs were excluded to maintain comparability across the two retail categories. We further address the potential selection bias introduced by this filtering rule through a hurdle-model-based robustness check, as detailed in Section 4.2. In this study, convenience stores are taken to represent light-asset formats, while large-scale supermarkets and shopping malls are chosen as representatives of capital-intensive formats. Retail location data are derived from the 2024 Gaode Map POI dataset, ensuring high accuracy and contemporaneity. After filtering, the final sample comprises 3,326 valid grid cells for light-asset retail and 893 for capital-intensive retail, as shown in Fig 2.

### 3.2. Data and variables calculation

Based on relevant literature and the characteristics of traditional retail [6,11], this study specifies two dependent variables corresponding to light-asset and capital-intensive formats, and a set of independent variables capturing six dimensions of location determinants: demographics, urban function, competition environment, traffic location, accessibility, and human perception. Table 1 summarizes the definitions, calculation methods, data sources, and data acquisition time for all variables. All measurements were conducted at the 500 m grid level using multi-source spatial data from Gaode Map, Baidu Map, Baidu Huiyan population heat map, and OpenStreetMap for the year 2024.

**Table 1:** Variable definitions and calculation method.

Category	Variables	Definition and calculation method	Data source	Period
**Dependent variables**				
Traditional retail location	Density of light-asset traditional retail	Density of convenience stores (num/grid)	Gaode Map	2024
	Density of capital-intensive traditional retail	Density of large-scale supermarkets and shopping malls (num/grid)		
**Independent variables**				
Demographics	Population density	Average population density in a week (num/grid)	Baidu Huiyan population heat map	2024
	Population mobility	The difference between the maximum and minimum daily population values in a week		
Urban function	Work place	Density of enterprises POIs (num/grid)	Gaode Map	2024
	Lodging	Density of lodging POIs (num/grid)		
	Medical facilities	Density of hospital, clinic, and health center POIs (num/grid)		
	Transit stops	Density of bus stops and subway stations road network		
Competition environment	Non-large supermarkets	Density of small and medium-sized supermarkets (num/grid)	Gaode Map	2024
	General markets	Density of tobacco and wine stores, fruit markets, and agricultural and sideline products markets (num/grid)		
Traffic location	Distance to bus stop	Distance to the nearest bus stops (m)	Gaode Map	2024
	Distance to subway	Distance to the nearest subway stations (m)		
Accessibility	Global integration	Accessibility of a space from all other spaces in the network	OpenStreetMap	2024
	Local integration	Accessibility of a space from spaces within 800 meters		
	Global choice	How often a space is used in shortest paths between all pairs of spaces		
	Local choice	How often a space is used in shortest paths between pairs of spaces within 800 meters		
	Road density	Density of road network (m /grid)		
Human perception	Vegetation	Proportion of greenery in the street-view image	Baidu Map	2024
	Beautiful	Residents’ perception of how beautiful the scene is		
	Depressing	Residents’ perception of how depressing the scene is		
	Lively	Residents’ perception of how lively the scene is		
	Safe	Residents’ perception of how safe the scene is		
	Wealthy	Residents’ perception of how wealthy the scene is		

#### 3.2.1. Traditional retail location.

To systematically compare the spatial patterns of light-asset and capital-intensive traditional retailers, the dependent variables are defined as follows: (1) The density of convenience stores, representing light-asset formats, is selected due to its ubiquity, small unit size, flexible operating models, and low barriers to entry. (2) The density of large-scale supermarkets and shopping malls, representing capital-intensive formats, includes supermarkets ranked in the 2023 China Top 100 Supermarket Chains and large-scale multi-category malls. These formats are characterized by their limited numbers, significant individual scale, and high capital requirements for both establishment and operation.

#### 3.2.2. Demographics.

Population density is widely recognized as a fundamental determinant of retail activity, with a long tradition of empirical research highlighting its impact on store performance and spatial distribution [16,44,45]. However, the traditional focus on static population counts may overlook the dynamic nature of urban retail demand [4]. Given that traditional retail is often more sensitive to foot traffic and transient flows than to residential population alone, this analysis further incorporates population mobility, calculated as the difference between the maximum and minimum daily population values within a week. Both metrics are derived from Baidu Huiyan population heat map data, which provide real-time, large-scale assessments of urban population dynamics.

#### 3.2.3. Urban function.

This study captures the multifaceted impact of urban functions on retailer spatial density by selecting four key variables, the densities of work places, lodging facilities, medical facilities, and transit stop, all of which are calculated based on the 2024 Gaode Map POI dataset. As shown in prior studies, these variables reflect core urban activities, population flows, and service accessibility, which directly shape consumer demand, daily mobility patterns, and the attractiveness of retail locations within each grid [46]. Incorporating these variables enables a comprehensive assessment of how different urban functions drive the spatial distribution of both light-asset and capital-intensive traditional retail.

#### 3.2.4. Competitive environment.

Competitive environment stands out as a crucial dimension that directly shapes the spatial strategies of traditional retail [6]. To assess the competitive environment, this study includes two variables: the density of small and medium-sized supermarkets, and the density of general markets such as tobacco and wine stores, fruit markets, and agricultural product markets. These variables were selected because they represent the primary sources of local retail competition faced by both light-asset and capital-intensive traditional retail. Including these indicators allows for a nuanced assessment of how different forms of proximate competition influence retailer location choices within urban grids.

#### 3.2.5. Traffic location.

To measure the accessibility of public transportation, we include two variables: distance to the nearest bus stop and distance to the nearest subway station. These indicators are selected because proximity to transit nodes significantly influences retail foot traffic, customer convenience, and overall store accessibility. Prior studies have shown that easy access to public transportation can enhance the attractiveness of retail locations, making these variables essential for understanding spatial patterns of different retail formats [11].

#### 3.2.6. Accessibility.

Five accessibility-related variables, global integration, local integration, global choice, local choice, and road density, are selected in the study to provide a comprehensive, multi-scale understanding of how urban spatial structure affects traditional retail location decisions. These variables reflect both overall and local accessibility as well as the connectivity and density of the road network. Prior research has shown that accessibility measures, such as network integration and road density, are significant predictors of retail success, influencing customer footfall, delivery efficiency, and spatial competition [3].

#### 3.2.7. Human perception.

**Fig 3:**
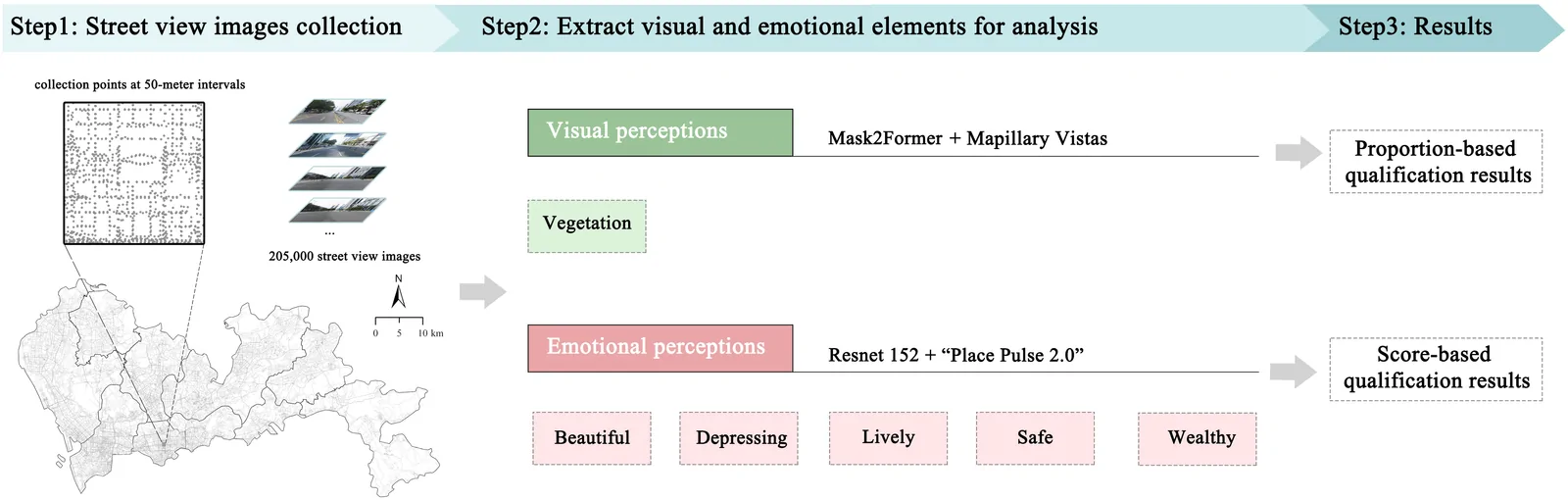
Analytical framework for human perception evaluation. Base map data from OpenStreetMap, available under the Open Database License (ODbL).

We incorporated six perceptual attributes, including vegetation, beautiful, depressing, lively, safe, and wealthy, to capture the human perception of street environments influencing retail location choice. These indicators represent residents’ perceptions of streetscapes, reflecting greenery coverage, aesthetic appeal, safety, vibrancy, affluence, and overall emotional tone. Evidence suggests that such perceptions significantly affect customer behavior and retail performance [11,38]. The streetscape feature extraction and analysis process are shown in Fig 3. We obtained 205,000 street-view images from Baidu Maps for Shenzhen. Vegetation was quantified using the Mask2Former segmentation framework trained on the Mapillary Vistas dataset, enabling accurate extraction of physical greenery. Emotional perceptions were derived from a ResNet‑152 model trained on the Place Pulse 2.0 dataset, producing scores for the five attributes.

To mitigate cross-cultural bias introduced by the Western-dominated Place Pulse 2.0 training dataset, we conducted a localized perception validation survey covering 15 representative street blocks across Shenzhen. These blocks were selected via stratified random sampling covering five major urban functional typologies (residential, central commercial, industrial, mixed-use and old urban village districts) to capture heterogeneous streetscape conditions across the city. A total of 80 local residents aged 18–65 completed anonymous on-site questionnaires. All five perceptual attributes (beautiful, lively, safe, depressing, wealthy) were evaluated using a 0–100 continuous visual analogue scale (VAS), matching the 0–100 output range of the ResNet152 perception model to enable direct numerical comparison. For correlation testing, all individual questionnaire responses were aggregated to generate block-level average human perception scores for each of the 15 sample blocks; we similarly averaged all street-view image model predictions within each corresponding block, and Pearson correlation coefficients were computed on these paired block-level aggregated values. Pearson correlation analysis demonstrates a significant positive correlation (r = 0.76, p < 0.001) between crowd-sourced local survey scores and model-generated perception values. Dimension-specific correlation results are as follows: beautiful (r = 0.81, p < 0.001), lively (r = 0.78, p < 0.001), depressing (r = 0.75, p < 0.001), safe (r = 0.73, p < 0.001), wealthy (r = 0.71, p < 0.001). All dimension-level correlations remain strongly statistically significant. This confirms that the pre-trained model’s outputs can reliably reflect Shenzhen residents’ subjective street evaluations despite its Western training base.

All on-site questionnaire procedures involving human participants followed institutional research governance standards. The participants were informed of the study objectives before the experiment and provided written informed consent. This study was approved by the Harbin Institute of Technology Medical Ethics Committee under ethics approval number HIT-2026049.

### 3.3. Data processing

#### 3.3.1. XGBoost model.

XGBoost is an advanced ensemble learning technique that iteratively constructs an additive model by combining multiple decision trees, each trained to correct the residual errors of its predecessors [47]. This gradient boosting framework enables high predictive accuracy and robustness against overfitting through integrated regularization methods. Compared to traditional gradient boosting approaches, XGBoost incorporates several computational optimizations, including parallel tree construction, cache-aware access, and efficient handling of sparse data, which significantly accelerate training and improve scalability. The model’s capacity to handle multicollinearity among features without feature elimination allows retention of all relevant predictors.

In this research, the dataset was randomly divided into training (80%) and testing (20%) subsets using a fixed random seed of 10 to ensure reproducibility. Model performance and generalizability were evaluated via five-fold shuffled cross-validation under the identical random state. The XGBoost model was implemented in Python via the xgboost package, with a unified full set of hyperparameters applied across all experimental groups. Fixed core structural parameters were set as n_estimators = 100, max_depth = 2, and random_state = 10. Additional calibrated hyperparameters include learning_rate = 0.1, subsample = 0.8, reg_alpha = 0.1, reg_lambda = 1.0, and min_child_weight = 3. All above hyperparameters were selected systematically via grid search cross-validation. A multi-dimensional candidate grid was predefined for learning rate, subsample ratio, regularization coefficients and minimum child weight. The grid search optimized the average five-fold cross-validation 𝑅^2^ as the core selection criterion; the hyperparameter combination achieving the highest cross-validated 𝑅^2^ was retained as the unified final configuration. The grid search pipeline shared identical data partitioning rules with formal model evaluation: shuffled five-fold KFold cross-validation with random state fixed at 10, ensuring consistent sample splitting throughout parameter tuning and subsequent model assessment.

Crucially, this full hyperparameter set was applied without modification across all experimental subgroups to guarantee fair comparative analysis: (1) two regression tasks for light-asset retail and capital-intensive retail; (2) three spatial grid resolutions (300 m, 500 m, 800 m) used for spatial unit sensitivity tests; (3) both stages of the hurdle robustness model, including the binary XGBoost classifier predicting retail presence within grids and the truncated XGBoost regressor estimating retail density for grids with positive retail counts. No hyperparameter adjustments were made for different subsets to avoid confounding comparative results caused by inconsistent model settings.

Model accuracy was assessed using 𝑅^2^, mean squared error (MSE), root mean squared error (RMSE), mean absolute error (MAE), and mean absolute percentage error (MAPE). The trained XGBoost model provides the foundation for subsequent interpretability analyses, such as SHAP values and PDP, facilitating an in-depth understanding of variable importance and nonlinear effects on traditional retail location selection.

#### 3.3.2. Shapley Additive exPlanations (SHAP).

SHAP is a unified framework for interpreting the output of machine learning models by quantifying the contribution of each feature to individual predictions [48]. Rooted in cooperative game theory, SHAP values are derived from the concept of Shapley values, which assign a fair distribution of a total payout to players based on their marginal contributions across all possible coalitions.

In the context of machine learning, each feature is treated as a “player” in a coalition, and the model prediction is considered the “payout.” The SHAP method computes the average marginal contribution of a feature by considering all possible subsets of features, thereby capturing the interactions and dependencies among features. This approach ensures that the feature attributions are consistent, locally accurate, and additive.

Formally, the explanatory model is articulated as:


$$\begin{array}{c} g\left(z^{\prime }\right)=\emptyset_{\text{0}}+\sum_{j=\text{1}}^{M}\emptyset_{j}{z^{\prime }}_{j} \end{array}$$ (1)

Here, g refers to the individual features under investigation in the next section. The vector $z^{\prime }\;\in {\left\{\;\text{0},\;\text{1}\right\}}^{M}$ describes all possible combinations (coalitions) of the M features, where each element indicates whether a feature is included. The feature importance, $\emptyset_{j}$, denotes the Shapley value attributed to feature j.

SHAP facilitates comprehensive interpretability by enabling global insights through feature importance rankings aggregated across the dataset, and local interpretability through visualization tools such as summary plots, dependence plots, and force plots. These visualizations help to reveal complex, nonlinear relationships between features and model outputs, as well as threshold or interaction effects that traditional linear methods may overlook.

#### 3.3.3. Partial Dependence Plots (PDP).

PDP serve as a visualization tool to assess the marginal impact of individual features on the predictions of machine learning models [49]. This method clarifies the associations between variables by means of a partial dependence function, formulated as follows:


$$\begin{array}{c} \hat{f_{xs}}\left(x_{S}\right)=E_{x_{C}}\left[\hat{f}\left(x_{S},x_{C}\right)\right]=\int \hat{f}(x_{S},x_{C})dP(x_{C}) \end{array}$$ (2)

Each variable S is associated with a unique partial dependence function $\hat{f_{s}}$, which computes the average value of the model output $\hat{f}$. Here, $x_{S}$ is held constant, while $x_{C}$ varies according to its marginal distribution dP($x_{C}$). It is important to note that both the model output $\hat{f}$ and the marginal distribution dP($x_{C}$) involve inherent uncertainties.

In this context, $\hat{f_{s}}$ represents the partial dependence function for variable S, which estimates the expected value of $\hat{f}$. when $x_{S}$ is fixed and$x_{C}$ fluctuates across its marginal distribution dP($x_{C}$). Given the uncertainties in both $\hat{f}$ and dP($x_{C}$), we use Monte Carlo integration to approximate Eq. (2) (Eq. 3):


$$\begin{array}{c} \hat{f_{S}}=\frac{\text{1}}{N}\sum_{i=\text{1}}^{N}\hat{f}(x_{S},x_{C_{i}}) \end{array}$$ (3)

This approximation utilizes feature values {$x_{C_{\text{1}}}$,  ..., $x_{C_{N}}$} extracted from the training dataset.

For multiclass classification tasks, PDP employs a one-vs-rest strategy, generating distinct plots for each class. While one-way PDP effectively reveals average marginal effects, it may mask localized interaction effects between variables. To capture these hidden complexities and explore multivariate relationships, we extend PDP to two-way analysis. This approach involves selecting pairs of variables related to the dependent variable and applying computational procedures consistent with Eq. (3) to quantify their joint effects.

Threshold positions along partial dependence curves were objectively identified via change-point detection for automatic extraction of slope inflection points. Bootstrap resampling was conducted at the grid-cell level with replacement over 1,000 replicates. The pre-trained XGBoost model was fixed for all bootstrap iterations; only partial dependence functions were recalculated on each resampled dataset to construct 95% confidence intervals for all estimated threshold breakpoints.

#### 3.3.4. Interpretative Structural Modeling (ISM).

ISM is a well-established methodology widely used in systems science and management research to elucidate complex systems by analyzing the interdependencies among their components. The ISM process begins with identifying key system elements filtered from the preceding XGBoost-SHAP analysis. To construct the Structural Self-Interaction Matrix (SSIM), ten domain specialists were invited to conduct pairwise contextual judgment of variable relationships. The expert panel comprises six tenured academic researchers focusing on urban retail geography and built environment analysis, together with four senior commercial planning practitioners who possess over eight years of professional experience in urban commercial spatial layout. We adopted a two-round Delphi-style elicitation protocol: in Round 1, all experts independently finished pairwise relationship assessments without cross-discussion to avoid mutual interference. After collecting all individual matrices, we sorted out all inconsistent variable relationship judgments; in Round 2, we held centralized group discussions to debate divergent items, and only finalized the unified SSIM after full group consensus was reached. We calculated the consistency coefficient of expert scoring results, specifically Kendall’s coefficient of concordance (Kendall’s W); the final consistency index reached 0.87, far above the acceptable threshold of 0.7 in Delphi research, which verifies that the panel’s collective judgment possesses sufficient inter-rater reliability and eliminates random subjective bias in constructing the adjacency matrix. Internal consistency tests were further performed on the finalized matrix to guarantee the stability of hierarchical partitioning results. This matrix is transformed into a reachability matrix using binary logic, enabling the determination of transitive directional dependencies among variables. Subsequent hierarchical partitioning organizes the elements into distinct levels, visualized as a directed graph. This hierarchical structure clearly delineates fundamental associational factors at the base level from dependent outcome variables at the apex, revealing both direct and indirect association pathways. By formalizing complex structural relationships among urban environmental predictors, ISM identifies critical leverage points and provides valuable decision support for retail spatial planning interventions amidst system complexity and uncertainty, particularly relevant for unpacking the layered spatial dynamics of light-asset and capital-intensive retail location strategies.

#### 3.3.5. Bayesian Network (BN).

**Fig 4:**
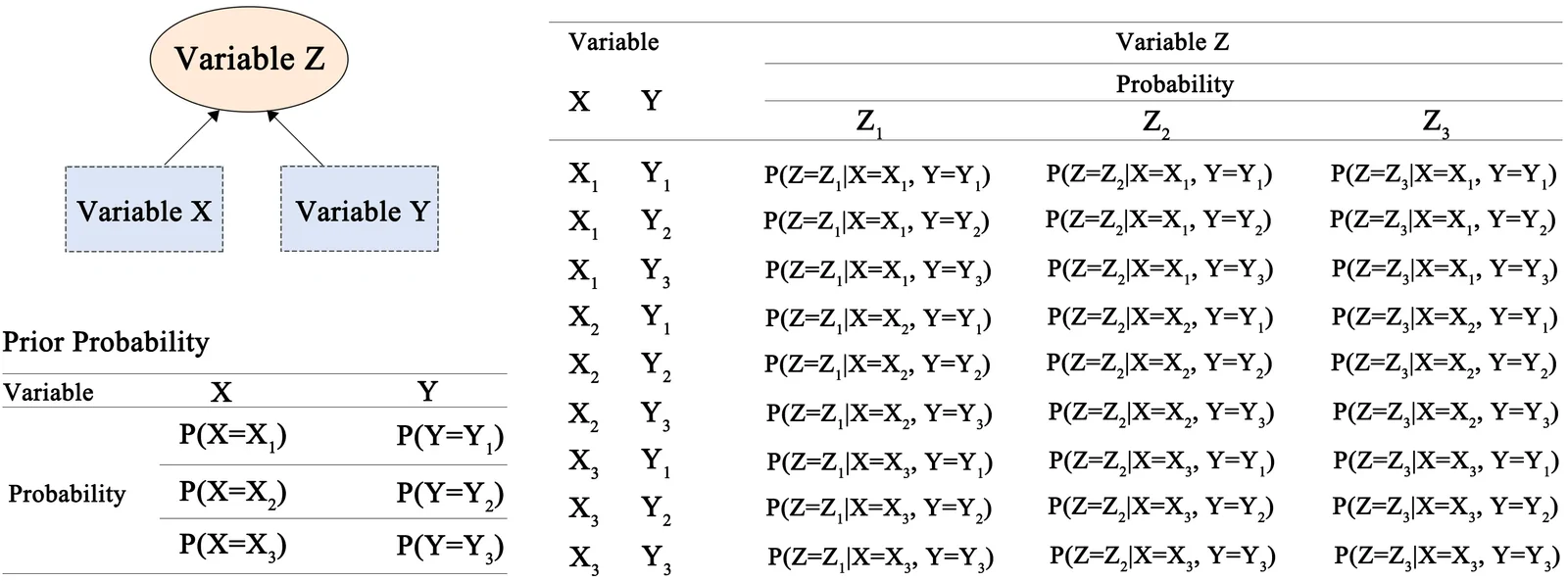
Bayesian Network structure and conditional probability table.

A Bayesian Network (BN) is a probabilistic graphical model that represents a set of variables and their conditional dependencies through a Directed Acyclic Graph (DAG) [50]. In the graph, nodes correspond to random variables and directed edges denote dependency relationships. The statistical influence between connected variables is encoded in conditional probability tables (Fig 4).

Given n variables $\{X_{\text{1}}$, $X_{\text{2}}$,  ..., $X_{n}\}$, the joint probability distribution factorizes according to the BN’s DAG structure:


$$\begin{array}{c} P\left(X_{\text{1}}{,X}_{\text{2}}{,\ldots ,X}_{n}\right)=\prod_{i=\text{1}}^{n}P\left(X_{i}|parent(X_{i})\right) \end{array}$$ (4)

where $parent(X_{i})$ represents the set of parent variables of $X_{i}$.

Bayesian inference updates beliefs in light of new evidence via Bayes’ theorem:


$$\begin{array}{c} P\left(V_{j}|D\right)=\frac{P\left(D|V_{j}\right)\cdot P(V_{j})}{\sum_{i=\text{1}}^{n}P\left(D|V_{i}\right)\cdot P(V_{i})} \end{array}$$ (5)

Here, $V_{j}$ denotes a hypothesis (e.g., a specific state of a target variable), D is observed evidence, $P(V_{j})$ is the prior probability, $P\left(D|V_{i}\right)$ is the likelihood, and $P\left(V_{j}|D\right)$ is the posterior.

In practice, continuous variables are discretized into non-overlapping intervals before network construction to meet the discrete CPT requirements. Once the structure and parameters are defined, BN enables forward and backward reasoning, sensitivity analysis, and probabilistic prediction of complex systems.

## 4. Results

### 4.1. Comparative spatial patterns of light-asset vs. capital-intensive traditional retail in Shenzhen

**Fig 5:**
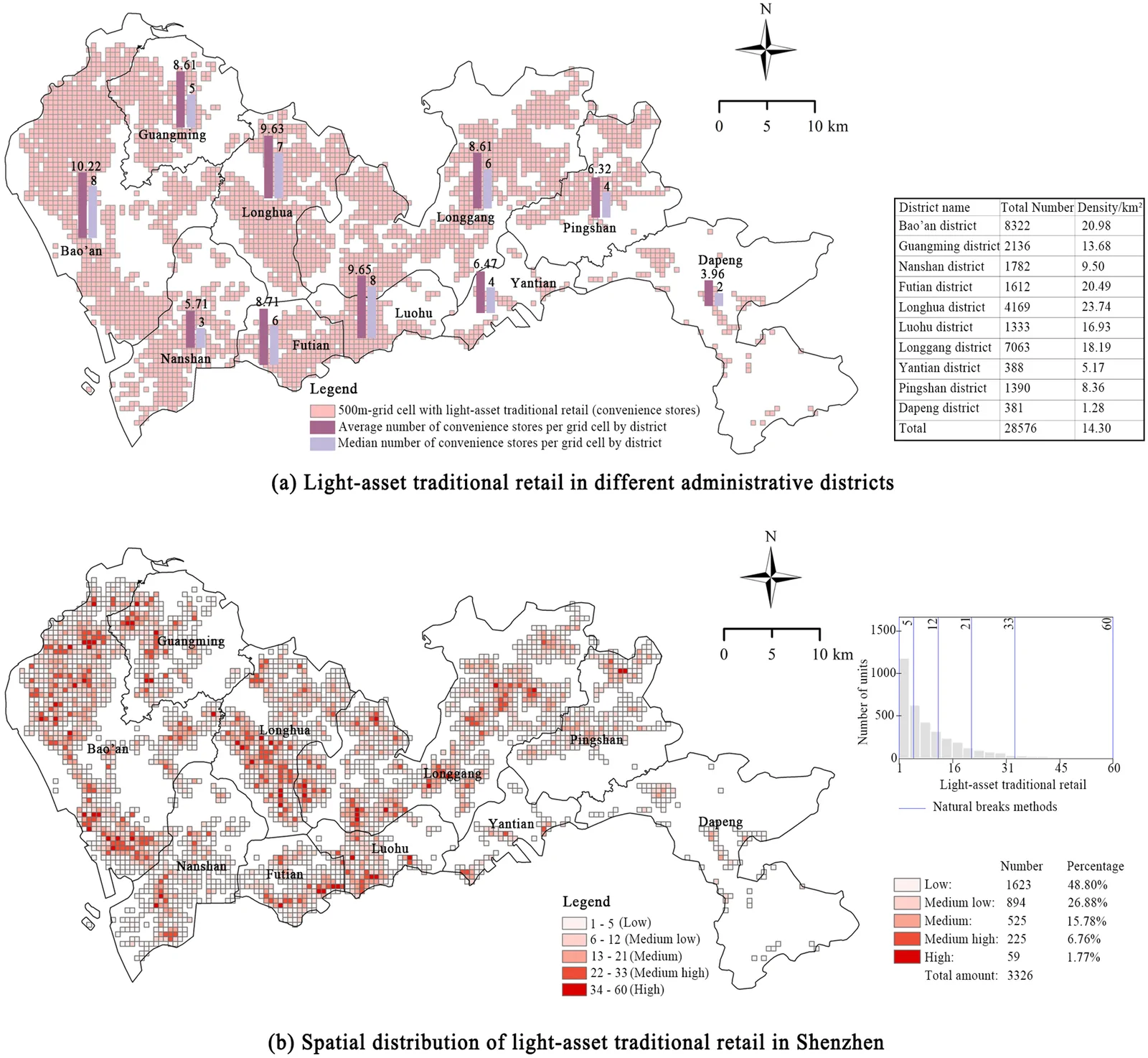
Spatial distribution and density of light-asset traditional retail: (a) Light-asset traditional retail in different administrative districts; (b) Density of light-asset traditional retail in Shenzhen. Base map data from OpenStreetMap, available under the Open Database License (ODbL).

**Fig 6:**
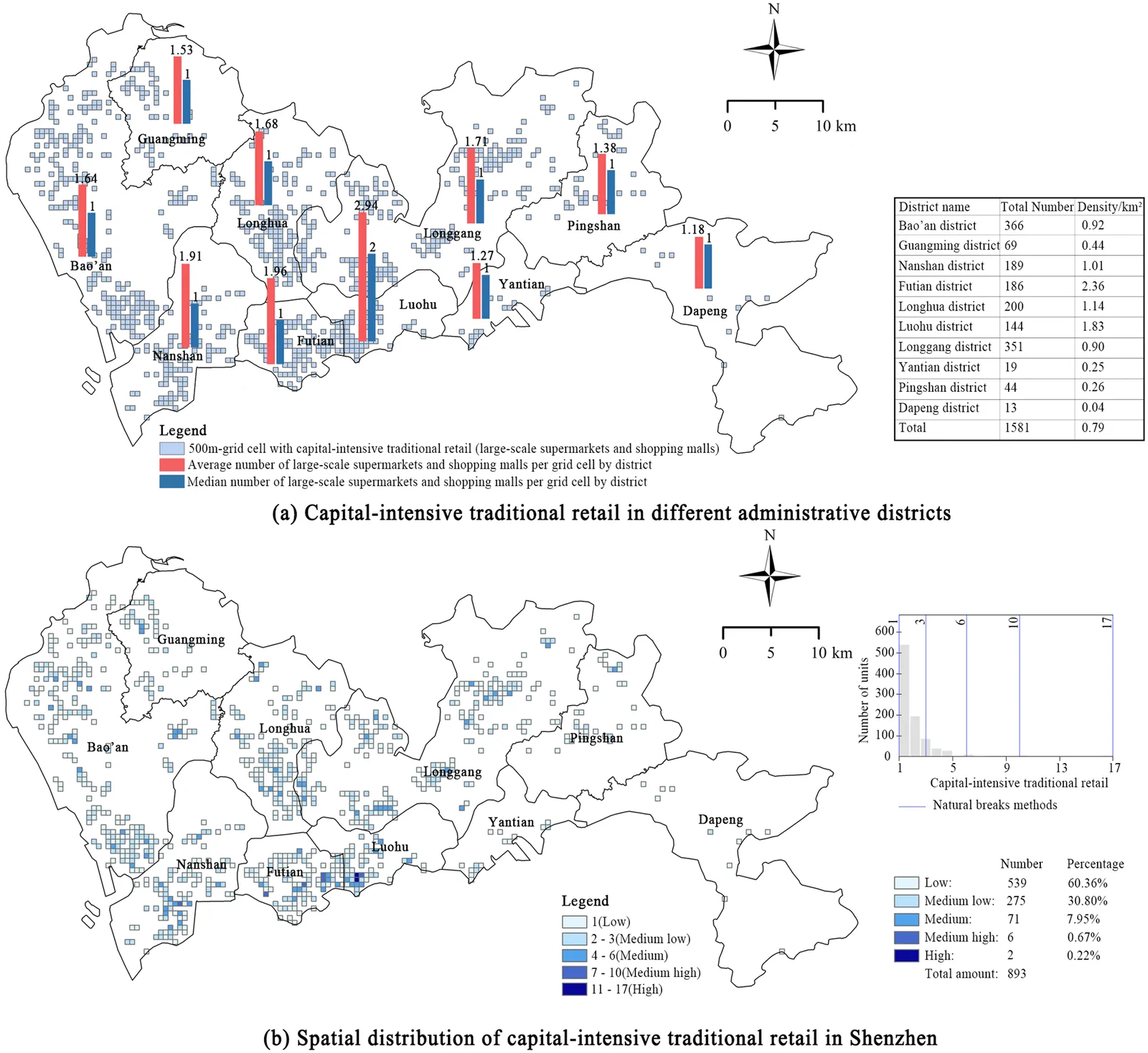
Spatial distribution and density of capital-intensive traditional retail: (a) Capital-intensive traditional retail in different administrative districts; (b) Density of capital-intensive traditional retail in Shenzhen. Base map data from OpenStreetMap, available under the Open Database License (ODbL).

Fig 5 and 6 demonstrate distinct spatial distribution patterns between light-asset traditional retail (convenience stores) and capital-intensive traditional retail (large-scale supermarkets and shopping malls) across Shenzhen. Light-asset traditional retail is widely dispersed throughout the city, with the highest average density observed in Bao’an (10.22 stores per grid) and Luohu (9.65 per grid), while peripheral districts such as Longhua and Longgang also maintain relatively high averages (9.63 and 8.61 per grid, respectively). Their notable presence in both central and peripheral areas reflects strong adaptability and low entry barriers. In contrast, capital-intensive traditional retail shows a markedly clustered distribution, primarily located in economically developed districts like Luohu, Nanshan and Futian, where average densities exceed 1.9 per grid. As illustrated in Fig 5(b), over 24.31% of the 500-meter grids host upwards of 13 convenience stores, underscoring the prolific presence of these compact, light-asset traditional retail that thrives through sheer numbers and dense clustering. In stark contrast, Fig 6(b) reveals that a vast majority, 91.16% of such grids accommodate no more than three large-scale supermarkets or shopping malls, entities that, while substantially larger in scale and capital investment, maintain a far more restrained spatial footprint.

The observed patterns can be attributed to the differing operational models: light-asset traditional retail requires smaller spaces and lower investments, allowing it to penetrate a wide range of urban contexts and meet daily consumer needs. Capital-intensive traditional retail, on the other hand, depends on larger customer catchments and substantial capital input, which limits its spatial presence to commercial hubs and high-demand areas. Urban planning policies and land use regulations further reinforce these distributional differences.

### 4.2. Visualization and comparison of variable contributions

To evaluate the determinants influencing the spatial density of light-asset and capital-intensive traditional retail, we tested multiple models including OLS and XGBoost. As shown in Table 2, XGBoost yields an R^2^ of 0.68 for light-asset retail and 0.42 for capital-intensive retail, versus OLS R^2^ values of 0.64 and 0.36. Meanwhile, the MAPE metrics reach 74.51% for light-asset retail and 45.49% for capital-intensive retail under XGBoost. It should be noted that MAPE tends to generate inflated values for count-type outcome variables. The grid-level retail density data contains numerous near-zero observations, which create tiny denominators and artificially magnify percentage error; accordingly, these raw MAPE figures should not be interpreted as direct evidence of weak model fit. Judging purely from absolute numerical standards, the capital-intensive R^2^ is modest and both models carry elevated MAPE levels; nevertheless, XGBoost still surpasses OLS across all R^2^, RMSE, MAE and MAPE benchmarks for both retail formats. Within the context of urban retail geospatial analysis, this level of model performance can still provide useful analytical insight. Retail density is jointly driven by quantifiable built-environment indicators and unmeasured micro-commercial attributes that cannot be fully extracted from open geospatial datasets. Although the model does not fully explain all variation in retail counts, the subsequent SHAP, PDP, ISM, and BN analyses focus primarily on relative variable importance, nonlinear response patterns, and hierarchical associations among predictors. These relational patterns may still offer meaningful and reasonably robust insights even under moderate overall model fit, thereby supporting the credibility of our comparative findings across the two retail categories. To address the potential sample selection bias caused by removing grid cells with zero retail POIs in the baseline model, we further constructed a two-stage XGBoost hurdle model covering the full set of 500 m grid samples (including all zero-density units). This framework separates two independent modeling tasks: a binary XGBoost classifier to predict the probability that a grid hosts retail outlets, and a truncated XGBoost regressor to estimate retail density only for grids with positive retail counts. We replicated the full SHAP variable importance ranking and extracted core nonlinear threshold effects based on hurdle model outputs. The results show highly consistent factor hierarchies, identical dominant influencing variables, and nearly overlapping critical threshold intervals compared with our baseline XGBoost model. This cross-verification confirms that excluding zero-retail grids does not distort the core comparative patterns between light-asset and capital-intensive retail derived in this study. Full performance metrics for the classification and truncated regression stages of the hurdle model, alongside side-by-side SHAP variable importance rankings against the baseline XGBoost, are documented in S7–S10 Tables of the Supporting Information, which numerically demonstrate high consistency in factor hierarchies and dominant influencing variables. Building on the calibrated XGBoost outputs, we applied SHAP to the trained XGBoost models to quantify and visualize the contribution of each variable to the prediction outcomes, enabling a fine-grained understanding of variable importance and directional impacts.

**Table 2:** Comparative analysis of model effectiveness.

Retailer type	Regression model	R²	RMSE	MAE	MAPE
Light-asset traditional retail	XGBoost	0.68	4.71	3.36	74.51
	OLS	0.64	5.03	3.60	85.34
Capital-intensive traditional retail	XGBoost	0.42	0.98	0.69	45.49
	OLS	0.36	1.02	0.76	49.21

**Fig 7:**
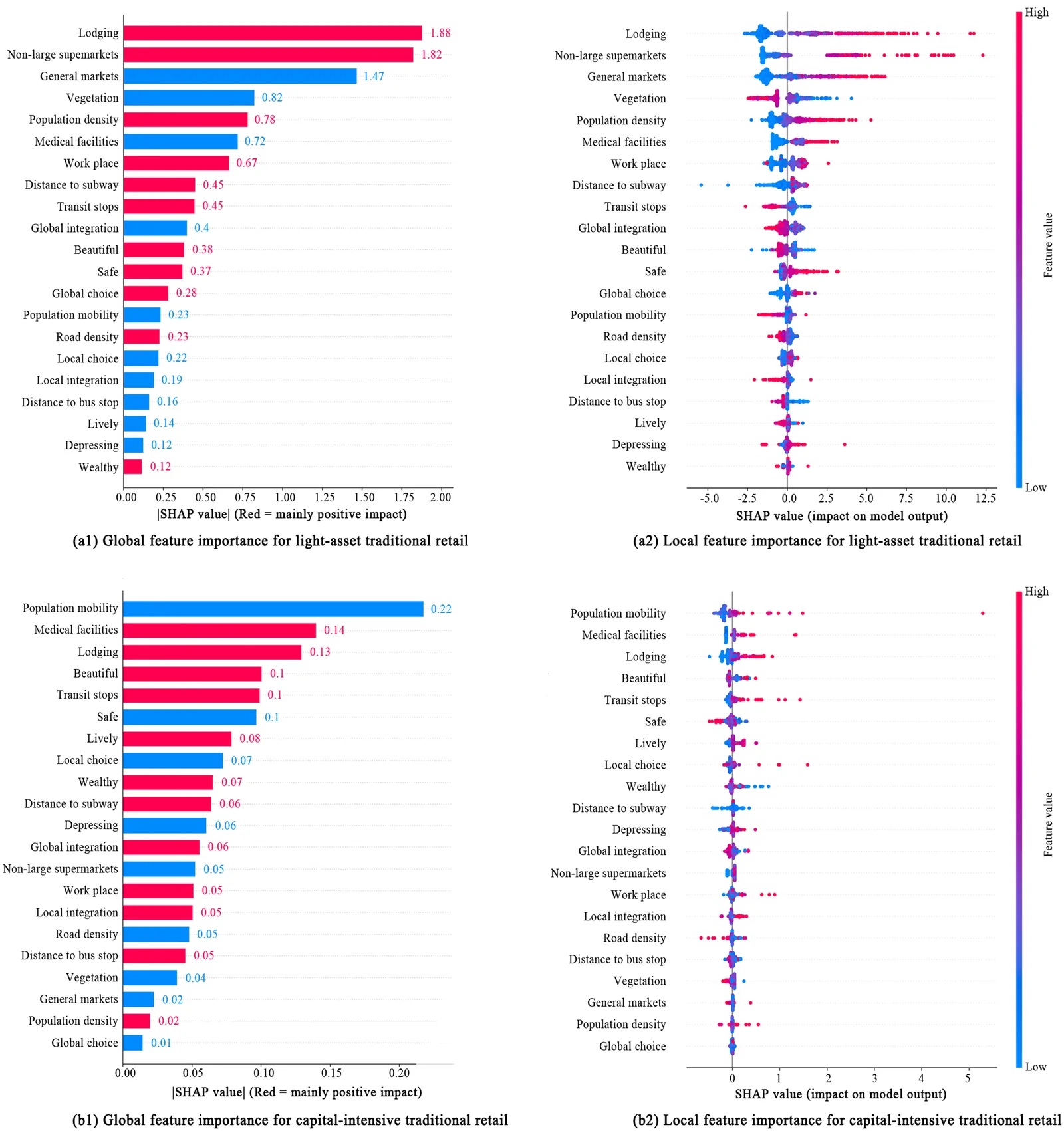
SHAP-derived feature contributions to retail location density: (a1) Global importance for light-asset traditional retail, (a2) Local importance for light-asset traditional retail, (b1) Global importance for capital-intensive traditional retail, and (b2) Local importance for capital-intensive traditional retail. Urban functional and competitive variables are more prominent for light-asset retail, whereas perceptual variables are more prominent for capital-intensive retail.

SHAP analysis revealed distinct influential factors for the two traditional retail types (Fig 7). For light-asset traditional retail, the three most positively impactful variables were lodging facilities’ density (SHAP = 1.88), non-large supermarkets’ density (1.82), and population density (0.78). These factors likely reflect the preference of light-asset traditional retail for areas with strong service infrastructure and moderate consumer traffic facilitating frequent but low-capital transactions. Conversely, the main negative influencers included general markets’ density (1.47), vegetation (0.82), and medical facilities’ density (0.72), possibly indicating that highly competitive or areas with dense greenery are less attractive for such retail. For capital-intensive traditional retail, medical facilities’ density (0.14), lodging facilities’ density (0.13), and beautiful (0.1) emerged as the top positive contributors. Negative influences for this retail included population mobility (0.22), safe (0.1), and local choice (0.07). Capital-intensive traditional retail tends to choose locations that are well-equipped and aesthetically pleasing but not overly “corridor-like,” as these areas can bring stable customer flow while enhancing the consumer experience and investment returns.

Examining variable categories further elucidates these patterns (Table 3). For light-asset traditional retail, the highest cumulative relative importance lies in urban function (31.18%), competition environment (27.64%), and human perception (16.42%), with competition environment (13.82%) leading average relative importance. In contrast, capital-intensive traditional retail prioritizes human perception (28.95%), urban function (27.56%), and accessibility (15.79%) cumulatively, while demographic factors (7.79%) top average importance. Notably, human perception exerts a strong impact of on both retailer types, highlighting the critical role of environmental perceptions in shaping traditional retail spatial density. Urban function also significantly influences both types, as mixed-use urban spaces support diverse customer flows and sustainable demand. However, competition environment greatly affects light-asset traditional retail due to their need for agility in competitive markets, whereas demographic stability and accessibility are vital for capital-intensive traditional retail to ensure long-term investment returns.

**Table 3:** Variable relative importance for density of light-asset vs. capital-intensive traditional retail.

Category	Variables	Light-asset traditional retail rel. imp.	Light-asset traditional retail cum. imp. (Avg.)	Capital-intensive traditional retail rel. imp.	Capital-intensive traditional retail cum. imp. (Avg.)
Demographics	Population density	6.58%	8.53% (4.27%)	1.30%	15.58% (7.79%)
	Population mobility	1.95%		14.28%	
Urban function	Work place	5.59%	31.18% (7.80%)	3.37%	27.56% (6.89%)
	Lodging	15.78%		8.49%	
	Medical facilities	6.04%		9.19%	
	Transit stops	3.77%		6.51%	
Competition environment	Non-large supermarkets	15.32%	27.64% (13.82%)	3.43%	4.91% (2.46%)
	General markets	12.32%		1.48%	
Traffic location	Distance to bus stop	1.35%	5.15% (2.58%)	2.99%	7.20% (3.60%)
	Distance to subway	3.80%		4.21%	
Accessibility	Global integration	3.34%	11.07% (2.21%)	3.66%	15.79% (3.16%)
	Local integration	1.60%		3.33%	
	Global choice	2.37%		0.93%	
	Local choice	1.84%		4.76%	
	Road density	1.92%		3.11%	
Human perception	Vegetation	6.91%	16.42% (2.74%)	2.57%	28.95% (4.83%)
	Beautiful	3.20%		6.60%	
	Depressing	1.03%		3.97%	
	Lively	1.17%		5.18%	
	Safe	3.13%		6.34%	
	Wealthy	0.98%		4.29%	

Note: rel. imp. = relative importance, cum. imp. = cumulative importance, Avg. = average importance. Relative importance represents each variable’s normalized mean absolute SHAP value. Cumulative relative importance is the sum of the relative importance values within each determinant category, whereas average relative importance is the cumulative value divided by the number of variables in that category. Higher values indicate stronger contributions to model predictions, but not the positive or negative direction of an effect.

Moderate to strong linear correlations are observed between conceptually related paired predictors, including accessibility indicators, transit metrics and emotional dimensions. The full Pearson correlation matrix is provided in S11 Table. Specifically, absolute correlation magnitudes peak at 0.42 for transit variables, 0.72 for the space-syntax Global choice–Local choice pair, and 0.71 for the perceptual Beautiful–Wealthy indicators. While XGBoost maintains stable predictive accuracy under multicollinearity, interrelated predictors will split total SHAP attribution values between one another. For this reason, SHAP importance metrics of variables belonging to the same correlated cluster should be interpreted as collective group contributions instead of discrete independent effects.

### 4.3. PDP analysis based on XGBoost model

**Fig 8:**
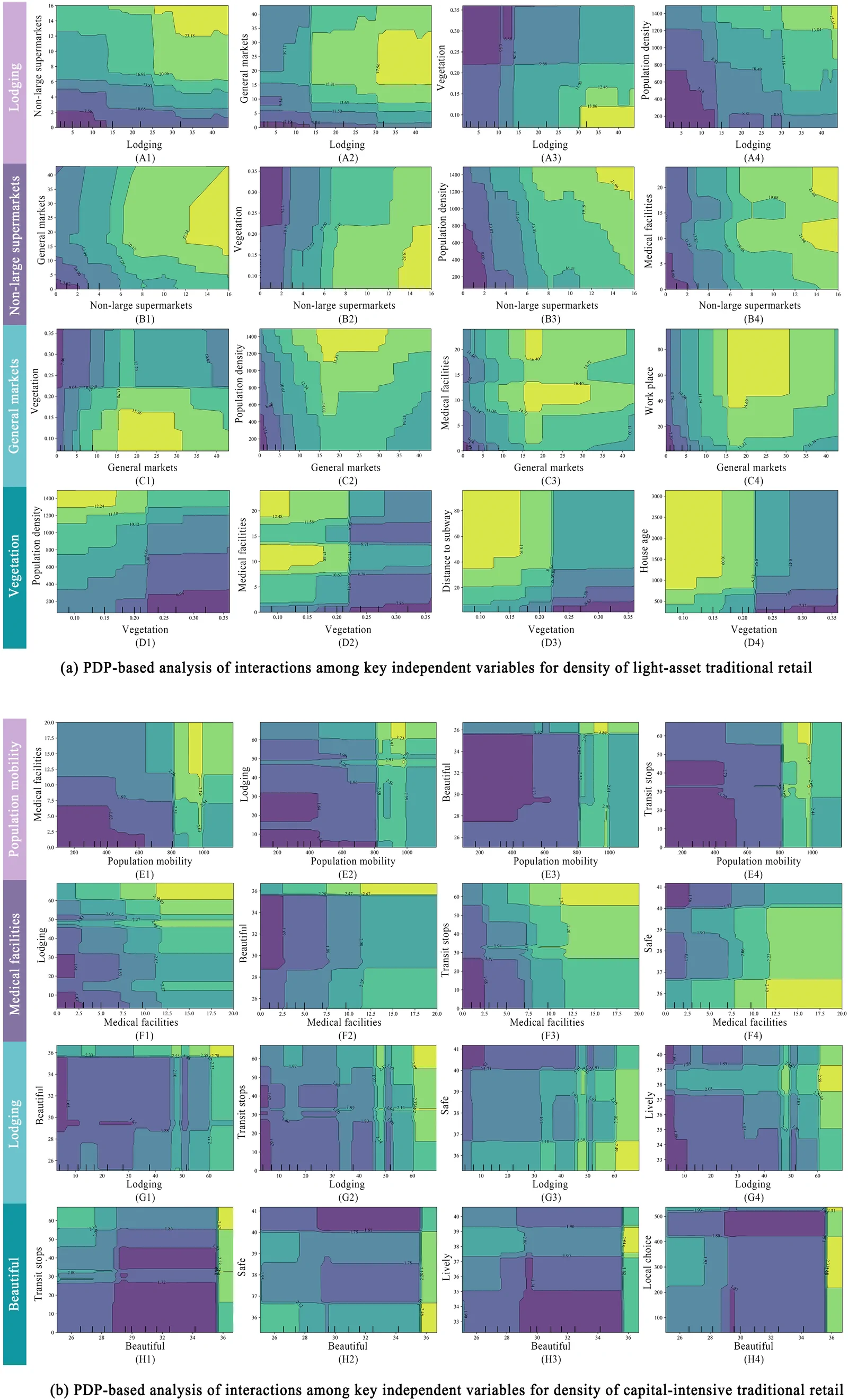
Interaction effects of urban environmental variables on traditional retail density derived from PDP analysis. (a) Light-asset traditional retail. (b)capital-intensive traditional retail. The results reveal moderate-density facility and competition synergies for light-asset traditional retail, but stronger threshold-dependent effects of population mobility and perceived environmental beauty for capital-intensive traditional retail.

To examine nonlinear associations and joint response patterns between key urban-environment variables and the predicted spatial density of the two retail formats, we conducted one-way and two-way partial dependence analyses for the most influential variables and selected theoretically relevant variable pairs identified from the SHAP results. In Fig 8, cooler colors indicate combinations associated with lower predicted retail density, whereas warmer colors indicate combinations associated with higher predicted retail density. The stability of the identified transition points was evaluated using 1,000 bootstrap resamples. All reported threshold cutoffs can be contextualized against the full empirical distribution of variables via S12 Table in Supporting Information. To improve readability, the main text reports only the principal nonlinear patterns and representative thresholds; the complete set of threshold estimates, interaction intervals, directional changes, and 95% bootstrap confidence intervals is provided in S13 Table of Supporting Information.

For light-asset traditional retail, lodging-facility density and non-large-supermarket density show generally positive marginal associations with predicted retail density, whereas vegetation shows an overall negative association. General-market density displays a clear nonlinear pattern: its marginal association is positive at low to moderate levels but becomes negative after reaching approximately 31 facilities per grid. This transition suggests that moderate concentrations of general markets may be associated with agglomeration and complementary-demand benefits, whereas higher concentrations correspond to market saturation and intensified local competition.

The two-way PDP results further show that the association between lodging-facility density and light-asset retail density becomes stronger when non-large-supermarket density or population density is also relatively high. General-market density and population density similarly reinforce the favorable association of non-large supermarkets with predicted retail density. By contrast, vegetation exhibits context-dependent joint patterns: although it has an overall negative marginal association, its relationship with retail density varies according to accessibility, population concentration, and the surrounding facility mix. Additional interval-specific patterns involving medical facilities and other service variables are reported in S13 Table of Supporting Information.

For capital-intensive traditional retail, lodging-facility density and medical-facility density show generally positive marginal associations with predicted retail density. Population mobility, however, exhibits an inverted-U pattern. Its association with retail density is favorable up to an intermediate range of approximately 900–1000, after which the marginal effect becomes negative. This finding indicates that moderate mobility is associated with a broader potential customer base, whereas highly transient population patterns may be less compatible with the stable catchment requirements of capital-intensive retail.

Perceived beauty also displays a threshold-dependent relationship. Its marginal association with capital-intensive retail density becomes positive only after the index reaches approximately 35.5. Two-way PDP results further indicate that higher levels of perceived beauty are jointly associated with higher predicted retail density when combined with population mobility, medical facilities, lodging facilities, or transit provision. Service-facility and transit variables likewise exhibit reinforcing joint patterns, suggesting that the concentration of capital-intensive retail is associated with the coexistence of accessibility, facility support, and environmental quality rather than with any single condition in isolation.

Other variables, including perceived liveliness, local street-network choice, lodging facilities, and perceived safety, show additional context-dependent interaction patterns. In particular, the negative conditional association involving perceived safety should not be interpreted as evidence that unsafe environments favor capital-intensive retail. It may instead reflect the broader land-use and morphological characteristics of locations that receive high safety scores but have relatively limited commercial intensity. The detailed interaction intervals and uncertainty estimates for these variables are presented in S13 Table of Supporting Information.

Overall, light-asset retail is characterized primarily by nonlinear competition and service-facility interactions, whereas capital-intensive retail displays stronger threshold-dependent associations with population stability and perceived environmental quality. These thresholds represent model-based empirical transition ranges identified within the Shenzhen sample. They should not be interpreted as causal effects, deterministic site-selection rules, or universally applicable planning cutoffs.

### 4.4. Model configuration and structure based on ISM and BN

Integrating ISM and BN enables a deeper understanding of the complex, hierarchical relationships among urban environmental variables, offering actionable insights into how these factors collectively drive the spatial dynamics of two traditional retail formats.

Based on SHAP analysis, fourteen key factors influencing the density of both light-asset and capital-intensive traditional retail were identified and subjected to ISM analysis. For density of light-asset traditional retail, the selected factors S_1_ to S_14_ represent lodging, non-large supermarkets, general markets, vegetation, population density, medical facilities, workplace, distance to subway, transit stops, global integration, beautiful, safe, global choice, and population mobility. For density of capital-intensive traditional retail, the variables X_1_ to X_14_ represent population mobility, medical facilities, lodging, beautiful, transit stops, safe, lively, local choice, wealthy, distance to subway, depressing, global integration, non-large supermarkets, and work place.

The adjacency matrices (Tables 4 and 5), which were constructed based on expert validation, encode the directly confirmed influences between factors (1 for presence, 0 for absence). These matrices serve as the basis for constructing the reachability matrices (Tables 6 and 7), which indicate whether an element can reach another, directly or indirectly, within the system (1 for reachable, 0 otherwise). Hierarchical partitioning (Tables 8 and 9) was performed by iteratively comparing the reachability and antecedent sets of each factor, extracting hierarchical levels until the structure for all elements was established.

**Table 4:** Adjacency matrix of variables influencing density of light-asset traditional retail.

Variables	S_1_	S_2_	S_3_	S_4_	S_5_	S_6_	S_7_	S_8_	S_9_	S_10_	S_11_	S_12_	S_13_	S_14_
**S**_**1**_	0	1	1	0	0	0	0	0	0	0	0	0	0	1
**S**_**2**_	0	0	0	0	0	0	0	0	0	0	0	0	0	1
**S**_**3**_	0	0	0	0	0	0	0	0	0	0	0	0	0	1
**S**_**4**_	0	0	0	0	0	0	0	0	0	0	1	1	0	0
**S**_**5**_	1	1	1	0	0	1	1	0	1	0	0	0	0	1
**S**_**6**_	1	1	1	0	0	0	0	0	0	0	0	0	0	1
**S**_**7**_	1	1	1	0	0	0	0	0	0	0	0	0	0	1
**S**_**8**_	0	0	0	0	0	0	0	0	0	0	0	0	0	0
**S**_**9**_	0	1	1	0	0	0	0	1	0	1	0	0	0	1
**S**_**10**_	0	0	0	0	0	0	0	0	0	0	0	0	0	0
**S**_**11**_	0	0	0	0	0	0	0	0	0	0	0	0	0	0
**S**_**12**_	0	0	0	0	0	0	0	0	0	0	0	0	0	0
**S**_**13**_	0	0	0	0	0	0	0	0	0	0	0	0	0	0
**S**_**14**_	0	0	0	0	0	0	0	0	0	0	0	0	0	0

**Table 5:** Adjacency matrix of variables influencing density of capital-intensive traditional retail.

Variables	X_1_	X_2_	X_3_	X_4_	X_5_	X_6_	X_7_	X_8_	X_9_	X_10_	X_11_	X_12_	X_13_	X_14_
**X**_**1**_	0	0	0	0	0	0	0	0	0	0	0	0	0	0
**X**_**2**_	1	0	1	0	0	0	0	0	0	0	0	0	1	0
**X**_**3**_	1	0	0	0	0	0	0	0	0	0	0	0	1	0
**X**_**4**_	0	0	0	0	0	0	0	0	0	0	0	0	0	0
**X**_**5**_	1	0	1	0	0	0	0	0	0	1	0	1	1	0
**X**_**6**_	0	0	0	0	0	0	0	0	0	0	0	0	0	0
**X**_**7**_	0	0	0	0	0	0	0	0	0	0	0	0	0	0
**X**_**8**_	0	0	0	0	0	0	0	0	0	0	0	0	0	0
**X**_**9**_	0	0	0	0	0	0	0	0	0	0	0	0	0	0
**X**_**10**_	0	0	0	0	0	0	0	0	0	0	0	0	0	0
**X**_**11**_	0	0	0	0	0	0	0	0	0	0	0	0	0	0
**X**_**12**_	0	0	0	0	0	0	0	0	0	0	0	0	0	0
**X**_**13**_	1	0	0	0	0	0	0	0	0	0	0	0	0	0
**X**_**14**_	1	0	1	0	0	0	0	0	0	0	0	0	1	0

**Table 6:** Reachable matrix of variables influencing density of light-asset traditional retail.

Variables	S_1_	S_2_	S_3_	S_4_	S_5_	S_6_	S_7_	S_8_	S_9_	S_10_	S_11_	S_12_	S_13_	S_14_
**S**_**1**_	1	1	1	0	0	0	0	0	0	0	0	0	0	1
**S**_**2**_	0	1	0	0	0	0	0	0	0	0	0	0	0	1
**S**_**3**_	0	0	1	0	0	0	0	0	0	0	0	0	0	1
**S**_**4**_	0	0	0	1	0	0	0	0	0	0	1	1	0	0
**S**_**5**_	1	1	1	0	1	1	1	1	1	1	0	0	0	1
**S**_**6**_	1	1	1	0	0	1	0	0	0	0	0	0	0	1
**S**_**7**_	1	1	1	0	0	0	1	0	0	0	0	0	0	1
**S**_**8**_	0	0	0	0	0	0	0	1	0	0	0	0	0	0
**S**_**9**_	0	1	1	0	0	0	0	1	1	1	0	0	0	1
**S**_**10**_	0	0	0	0	0	0	0	0	0	1	0	0	0	0
**S**_**11**_	0	0	0	0	0	0	0	0	0	0	1	0	0	0
**S**_**12**_	0	0	0	0	0	0	0	0	0	0	0	1	0	0
**S**_**13**_	0	0	0	0	0	0	0	0	0	0	0	0	1	0
**S**_**14**_	0	0	0	0	0	0	0	0	0	0	0	0	0	1

**Table 7:** Reachable matrix of variables influencing density of capital-intensive traditional retail.

Variables	X_1_	X_2_	X_3_	X_4_	X_5_	X_6_	X_7_	X_8_	X_9_	X_10_	X_11_	X_12_	X_13_	X_14_
**X**_**1**_	1	0	0	0	0	0	0	0	0	0	0	0	0	0
**X**_**2**_	1	1	1	0	0	0	0	0	0	0	0	0	1	0
**X**_**3**_	1	0	1	0	0	0	0	0	0	0	0	0	1	0
**X**_**4**_	0	0	0	1	0	0	0	0	0	0	0	0	0	0
**X**_**5**_	1	0	1	0	1	0	0	0	0	1	0	1	1	0
**X**_**6**_	0	0	0	0	0	1	0	0	0	0	0	0	0	0
**X**_**7**_	0	0	0	0	0	0	1	0	0	0	0	0	0	0
**X**_**8**_	0	0	0	0	0	0	0	1	0	0	0	0	0	0
**X**_**9**_	0	0	0	0	0	0	0	0	1	0	0	0	0	0
**X**_**10**_	0	0	0	0	0	0	0	0	0	1	0	0	0	0
**X**_**11**_	0	0	0	0	0	0	0	0	0	0	1	0	0	0
**X**_**12**_	0	0	0	0	0	0	0	0	0	0	0	1	0	0
**X**_**13**_	1	0	0	0	0	0	0	0	0	0	0	0	1	0
**X**_**14**_	1	0	1	0	0	0	0	0	0	0	0	0	1	1

**Table 8:** Intersection analysis of reachable and antecedent sets for density of light-asset traditional retail.

Variables	The reachable set	The intersection set
S_1_	S_1_, S_2_, S_3_, S_14._	S_1_
S_2_	S_2_, S_14._	S_2_
S_3_	S_3_, S_14._	S_3_
S_4_	S_4_, S_11_, S_12._	S_4_
S_5_	S_1_, S_2_, S_3_, S_5_, S_6_, S_7_, S_8_, S_9_, S_10_, S_14._	S_5_
S_6_	S_1_, S_2_, S_3_, S_6_, S_14._	S_6_
S_7_	S_1_, S_2_, S_3_, S_7_, S_14._	S_7_
S_8_	S_8_	S_8_
S_9_	S_2_, S_3_, S_8_, S_9_, S_10_, S_14._	S_9_
S_10_	S_10_	S_10_
S_11_	S_11_	S_11_
S_12_	S_12_	S_12_
S_13_	S_13_	S_13_
S_14_	S_14_	S_14_

**Table 9:** Intersection analysis of reachable and antecedent sets for density of capital-intensive traditional retail.

Variables	The reachable set	The intersection set
X_1_	X_1_	X_1_
X_2_	X_1_, X_2_, X_3_, X_13_	X_2_
X_3_	X_1_, X_3_, X_13_	X_3_
X_4_	X_4_	X_4_
X_5_	X_1_, X_3_, X_5_, X_10_, X_12_, X_13_	X_5_
X_6_	X_6_	X_6_
X_7_	X_7_	X_7_
X_8_	X_8_	X_8_
X_9_	X_9_	X_9_
X_10_	X_10_	X_10_
X_11_	X_11_	X_11_
X_12_	X_12_	X_12_
X_13_	X_1_, X_13_	X_13_
X_14_	X_1_, X_3_, X_13_, X_14_	X_14_

For density of light-asset traditional retail, the hierarchy consists of six levels: (1) density of light-asset traditional retail; (2) distance to subway, global integration, beautiful, safe, global choice, and population mobility; (3) vegetation, non-large supermarkets, general markets; (4) lodging, transit stops; (5) medical facilities, work place; (6) population density. For density of capital-intensive traditional retail, the structure includes five levels: (1) density of capital-intensive traditional retail; (2) beautiful, safe, lively, local choice, population mobility, wealthy, distance to subway, depressing, global integration; (3) non-large supermarkets; (4) lodging; (5) workplace, transit stops, medical facilities. The ISM hierarchy illustrates the pathways through which urban environment variables affect retail density.

**Fig 9:**
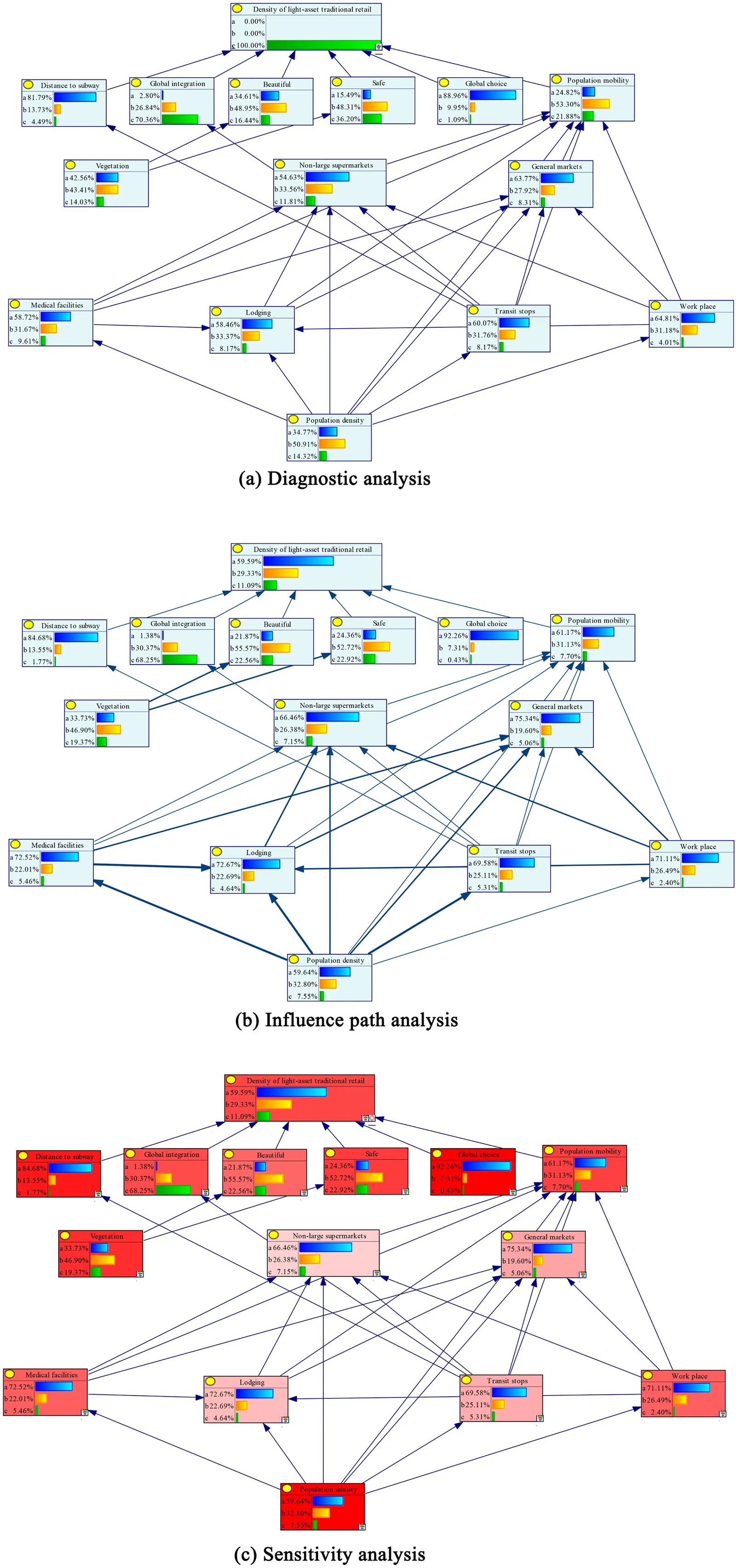
BN-based evaluation model for density of light-asset traditional retail. (a) Diagnostic analysis. (b) Influence path analysis. (c) Sensitivity analysis. The results reveal multiple entry points and interconnected pathways underlying light-asset retail clustering.

To ensure computational efficiency and scale invariance, variables were discretized using the Jenks natural breaks method. All continuous predictors were split into three quantile-based non-overlapping intervals via Jenks natural breaks binning. Each BN directed acyclic graph was fully constrained by the hierarchical digraph generated from ISM, without any data-driven structure learning. Conditional probability tables for all nodes were estimated using empirical frequency counts from the discretized grid sample dataset. BN inference results are acknowledged to be sensitive to discretization binning schemes, and all pathway interpretations below account for this limitation. Spatial datasets for both retail types were processed in GeNIe for model training. Each Bayesian network model comprises 15 nodes, each discretized into three states, with state probabilities derived from historical data, including prior and conditional probabilities. As shown in Fig 9, integrating the results from GeNIe-based diagnostic analysis, influence path analysis, and sensitivity analysis, two primary pathways were identified as having the strongest impact on density of light‑asset traditional retail. The first pathway operates predominantly through perceptual factors: increased vegetation influences perceived environmental qualities such as safe and beautiful, which subsequently stimulate the clustering of light‑asset traditional retail. This effect can be attributed to the fact that aesthetically pleasing and perceived-safe environments not only attract pedestrian flows but also encourage longer dwell times, thereby increasing the potential customer base and improving the commercial viability of such retailers. The second pathway functions through objective urban environment factors: higher population density increases the density of transit stops, reducing the average distance to metro stations and improving accessibility. Enhanced accessibility facilitates greater consumer mobility and convenience, which are critical for the frequent and spontaneous visits typically associated with light‑asset traditional retail formats.

**Fig 10:**
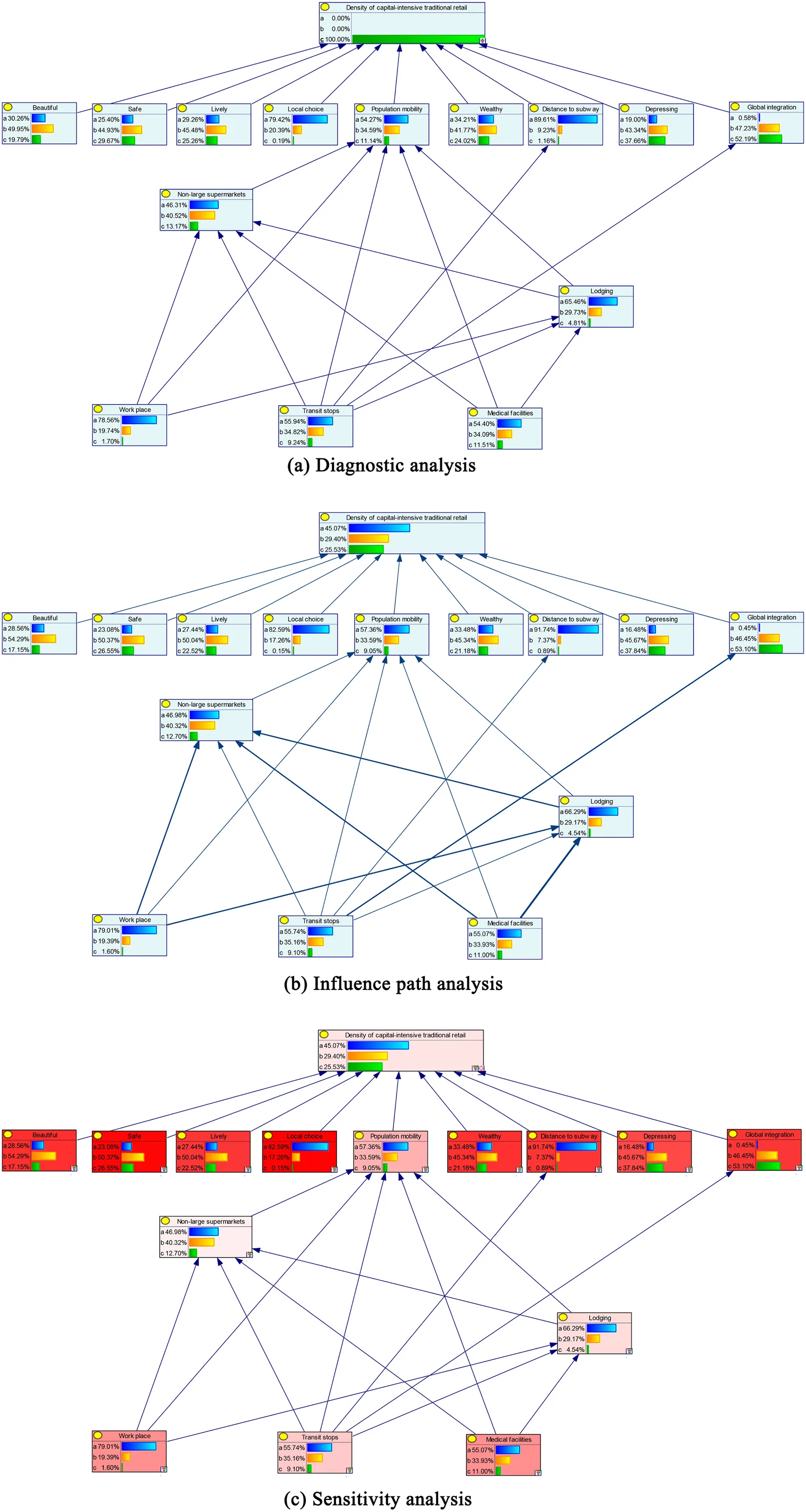
BN-based evaluation model for density of capital-intensive traditional retail. (a) Diagnostic analysis. (b) Influence path analysis. (c) Sensitivity analysis. The results reveal a relatively sequential and hierarchical pathway underlying capital-intensive retail clustering.

As depicted in Fig 10, for density of capital‑intensive traditional retail, in addition to the direct influence of subjective factors such as beauty, safety, liveliness, wealthiness, and the absence of depressing features, a prominent objective factor pathway was identified. Higher medical facility density promotes the growth of lodging facilities, which in turn increases the density of non‑large supermarkets, subsequently influencing population mobility. This chain of effects ultimately fosters an environment conducive to capital‑intensive traditional retail. The underlying mechanism lies in the complementary relationship between these facilities: medical centers and lodging attract steady flows of visitors and citizens, supermarkets provide immediate consumption options, and together they generate sustained foot traffic and diversified demand, both essential for the economic feasibility of high‑investment retail operations.

The hierarchical stratification derived from ISM clearly distinguishes exogenous core associational factors and intermediate mediating variables for two retail formats. For light-asset retail, population density sits at the bottom foundational layer, with multiple parallel intermediate mediating paths (service facilities, street perception) leading to retail density, which matches its scattered, multi-source agglomeration characteristics. By contrast, capital-intensive retail presents a single sequential upstream chain rooted in medical and lodging facilities, reflecting its reliance on complete supporting commercial clusters before large-scale investment entry. The Bayesian network further quantifies the probabilistic strength of each ISM hierarchical pathway through conditional probability tables. The integrated ISM-BN framework does not merely output static correlation hierarchies; it realizes dynamic scenario reasoning by simulating changes of single or multiple urban environment variables, which provides quantifiable planning reference rather than qualitative description alone. Notably, BN pathway inference is sensitive to the discretization binning scheme adopted here, and all reported association pathways are interpreted with this limitation in mind.

## 5. Discussion

### 5.1. Nonlinear, interactive, and hierarchical mechanisms shaping spatial density of traditional retail

This study advances the understanding of retail location dynamics by demonstrating that the spatial patterns of light‑asset and capital‑intensive formats are shaped not by linear correlations, but by nonlinear responses, synergistic interactions, and hierarchical dependencies among urban environment variables. The superior performance of XGBoost over OLS confirms that conventional linear models used in previous research overlook critical threshold effects and context‑contingent shifts, potentially leading to misinterpretation of locational logic [11,51].

A key insight is that the same environmental factor can act as either an enabler or a constraint depending on retailer format and local saturation level. This format-based divergence in threshold and interaction mechanisms can be systematically interpreted through classical retail location theories including Central Place Theory and retail agglomeration differentiation theory. The core internal driver lies in fundamental gaps in capital input, operation cycle, consumer positioning and catchment scale between the two retail types. Light-asset convenience stores are short-cycle, low-cost daily retail targeting scattered resident spontaneous consumption, thus forming low-competition tolerance thresholds and weak dependence on streetscape aesthetics. By contrast, capital-intensive supermarkets and malls feature long depreciation cycles, huge land and decoration expenditures, and target comprehensive, planned consumption; they therefore impose strict perceptual quality cutoffs and demand stable resident groups instead of highly mobile transient populations. Competitive density produces positive spillovers for light‑asset retailers up to a moderate threshold, after which congestion effects erode viability, in line with prior studies [52]. Similarly, population mobility also has a format-specific effect: capital-intensive retail benefits from moderate mobility but becomes less strongly associated with locations characterized by excessive population turnover; excessive mobility undermines the operational stability prized by capital‑intensive ventures, thus extending and deepening the conclusions of relevant study [3]. These nonlinear tipping points highlight the need to frame planning interventions around “functional ranges” rather than monotonic assumptions.

Interaction effects detected through two‑way PDP analysis reveal that retailer clustering is seldom the additive result of single variables. Light‑asset formats profit from combinations of functional service proximity and perceptually pleasant surroundings, which jointly lengthen dwell time and stimulate impulse purchases. Capital‑intensive formats, by contrast, require convergence between stable service anchors such as medical, lodging and high‑quality aesthetic environments before density gains emerge, suggesting a dependency on both structural stability and brand‑aligned imageability. Such findings refine classical retail location theory by showing that operational models condition how built and perceived attributes are filtered and prioritised.

The ISM‑BN integration further reframes retailer density as the outcome of structured, multi‑layered correlative chains. For light‑asset formats, clustering can be initiated via multiple “entry points”, either high accessibility through transit and population cores or perceptually enhanced environments that stimulate footfall. This adaptability contrasts with the sequential, dependency‑heavy pathways of capital‑intensive formats, which often hinge on the prior accumulation of supportive anchors before complementary facilities and high‑quality consumer flows can materialise. In both cases, deep‑layer spatial enablers such as accessibility and functional diversity operate indirectly, channelling influence through mid‑tier perceptual and competitive mediators.

By articulating these systemic architectures, the present study moves the debate beyond identifying “which factors matter” to explaining “how and under what conditions they matter.” For urban planners, this implies that optimising retail vitality is less about maximising any single metric and more about orchestrating the timing, balance, and co‑occurrence of supporting conditions. Policies that respect nonlinear thresholds, foster productive facility synergies, and sequence infrastructure, perception, and competition in format‑appropriate ways are better positioned to sustain diverse and resilient retail ecosystems.

### 5.2. Human perception as a core driver of traditional retail location choice

While the urban environment’s influence on retailer location choice has been widely acknowledged, prior research has predominantly emphasized objective urban features [3,6]. Far less systematic attention has been devoted to understanding how micro-scale street environments, and the combined effects of objective and subjective human perceptions, jointly shape the spatial density of different traditional retail. This study addresses this gap by integrating both dimensions, drawing on multi-sourced urban datasets and advanced computational modeling.

Our findings underscore that human perception metrics are not peripheral, but central determinants of spatial patterns in both light‑asset and capital‑intensive traditional retail, albeit with differing modes of influence. SHAP results reveal that perception‑based indicators account for 16.42% of cumulative importance in light‑asset formats and nearly 29% in capital‑intensive formats, ranking as the most influential category for the latter. This magnitude exceeds or rivals that of purely objective factors, indicating that how an area is experienced can be as decisive for retailer agglomeration as its functional accessibility or demographic base.

The XGBoost‑PDP analysis further confirms that perceptual attributes exert clear threshold effects. For capital‑intensive retail, perceived beauty becomes positively associated with retail density only after a relatively high level of environmental quality is reached. Below this threshold, perceptions may deter investment, even if objective infrastructure is favourable. Light‑asset retail shows a different pattern: perceptual factors such as beautiful or safe do contribute, but their greatest value lies in synergistic interactions with functional variables.

The ISM–BN modelling further clarifies the probabilistic association pathways linking perception and retail density. For light‑asset formats, perception acts as an intermediate amplifier: creating an environment where pedestrians feel safe and visually stimulated not only attracts more flows but also prolongs dwell time, which is particularly valuable for frequent‑purchase, convenience‑oriented businesses. For capital‑intensive formats, perceptions operate more directly as top‑tier nodes in the urban environment hierarchy, shaping market attractiveness even before accessibility or facility mix come into play. This reflects their strategic emphasis on long‑term brand equity, aesthetic alignment, and stability of return on investment.

Domestic urban studies utilizing Chinese street view imagery have advanced quantitative measurement of street greenery and multi-dimensional perceptual aesthetics, and preliminarily linked perceived street quality to urban vitality [36,37,53]. Nevertheless, existing research mostly concentrates on general spatial patterns of perceptual indicators across cities, without unpacking heterogeneous threshold effects of human-centered street perceptions for distinct retail formats. While prior scholarship uniformly aggregates all offline retail outlets for analysis, this study further differentiates light-asset and capital-intensive traditional retail, revealing divergent sensitivity thresholds to street beauty, liveliness and green coverage. It therefore supplements targeted comparative empirical evidence missing from domestic retail location research, and extends street-view-based perceptual analysis to format-specific commercial spatial research.

For urban planners and policy-makers, these results suggest that strategies aimed at enhancing retail vitality cannot rely solely on improving physical infrastructure or demographic targeting. Instead, they must also engineer and sustain favourable perception environments, by investing in streetscape aesthetics, public safety measures, and programming that fosters urban vibrancy. For light‑asset retail, such interventions can magnify the returns of functional clustering; for capital‑intensive retail, they may be a prerequisite for location viability. Integrating perception metrics into planning models thus provides a more complete and predictive framework for sustaining diverse retail ecosystems. From the perspective of retail format segmentation theory, this study supplements a neglected dimension of asset intensity division to existing perceptual location research. Prior retail geography studies mostly treat physical retail as a unified category and ignore that streetscape perception’s threshold constraints are format-specific. Our comparative results complement Central Place Theory by revealing that low-order retail only takes perceived environment as auxiliary attraction, while high-order large-scale commercial facilities regard high aesthetic scores as a necessary precondition for site selection, further enriching the micro environmental interpretation system of classical retail spatial theories.

### 5.3. Policy implications

The results support a format-specific and threshold-sensitive approach to retail planning. Rather than maximizing commercial density, accessibility, population flow, or environmental quality independently, planning interventions should consider whether these conditions fall within locally appropriate ranges and whether complementary urban functions are jointly present. The empirical transition ranges identified in this study can support local diagnosis and scenario evaluation, but they should not be treated as universal or deterministic regulatory cutoffs.

For light-asset retail, neighborhood planning should support moderate commercial agglomeration while avoiding excessive concentrations of homogeneous outlets. Convenience stores benefit from proximity to residential populations, lodging functions, workplaces, transit services, and complementary small-scale businesses, but excessive local competition may reduce the viability of additional stores. Planning should therefore emphasize functional complementarity and tenant diversity rather than simply increasing outlet numbers. Preserving fine-grained street-front premises and affordable small commercial spaces can also help maintain neighborhood accessibility, grassroots entrepreneurship, and the adaptive capacity of light-asset retail.

Streetscape interventions for light-asset retail should balance environmental quality with commercial visibility and pedestrian access. Greening can improve thermal comfort and walking experience, but its design should avoid obscuring storefronts, interrupting pedestrian connections, or reducing usable commercial frontage. The relevant implication is not to restrict urban vegetation, but to coordinate greenery, frontage design, signage visibility, and pedestrian circulation in neighborhood commercial streets.

For capital-intensive retail, site evaluation should consider the joint presence of stable demand, supportive service facilities, multimodal accessibility, and high-quality public environments. High pedestrian or population mobility alone does not necessarily provide the stable catchment required by large supermarkets and shopping malls. Similarly, streetscape improvements may have limited commercial effects when basic transport connections, facility networks, and market demand are insufficient. Large commercial land releases should therefore be preceded by integrated assessment of catchment stability, surrounding functions, accessibility, and experiential environmental quality.

The hierarchical associations identified by the ISM–BN analysis also suggest that retail-supporting interventions should be coordinated and appropriately sequenced. In prospective commercial hubs, public authorities should align service facilities, transport provision, public-space improvements, and commercial land supply rather than relying on isolated infrastructure projects. However, the model does not imply that a single fixed development sequence will be optimal in every context; the appropriate order should be calibrated to local land-use conditions, existing facility networks, and market demand.

At the citywide level, a dual-track policy framework can help balance inclusiveness and economic competitiveness. Fine-grained commercial spaces should be retained in residential and mixed-use neighborhoods to support light-asset retail and everyday access to essential goods. At the same time, suitable sites with stable catchments, sufficient infrastructure, and high-quality public environments should be reserved or regenerated for capital-intensive retail. Because the numerical thresholds identified in Shenzhen may vary with urban morphology, data resolution, consumer behavior, and local market conditions, their application in other cities requires local recalibration and additional validation.

### 5.4. Research limitations and future research directions

Four structural limitations merit acknowledgment. First, the framework models location patterns primarily as a function of urban environmental factors, without explicitly incorporating potential strategic interactions among retailers such as first‑mover deterrence and coalition siting. Such game‑theoretical positioning data remain commercially sensitive and are inaccessible to public research, necessitating confidential, long‑term collaboration with industry actors to reconstruct authentic decision chains. Secondly, the ResNet-152 model for street perception relies on the Place Pulse 2.0 dataset, which primarily captures Western urban streetscapes. Although we performed localized questionnaire calibration to align model outputs with Shenzhen residents’ subjective experience, regionally optimized visual perception models trained on Chinese street-view imagery would further eliminate cross-cultural deviations in follow-up research. Thirdly, this study’s variable pool does not fully incorporate several institution- and market-based core determinants that exert decisive impacts on retail layout, including block-level land prices, urban zoning regulations, commercial lease terms, private developer investment strategies, and historical land-use trajectories. Such institutional and commercial data are not publicly available at a citywide scale in Shenzhen, and unified open geospatial databases lack standardized records of these indicators. Future work could cooperate with local urban planning bureaus and real estate developers to integrate these omitted predictors and further enhance model explanatory power. Fourth, although the integrated XGBoost–SHAP–PDP–ISM–BN framework improves the interpretability of complex retail-location mechanisms, some uncertainty in the ISM–BN reasoning process remains unavoidable when modelling urban systems. In this study, such uncertainty was reduced through a two-round Delphi-based expert elicitation process, inter-rater consistency testing, and internal consistency checks; however, the ISM structure still represents a simplified abstraction of inter-variable relationships, while BN inference remains probabilistic and may be affected by data quality, discretization, and finite-sample estimation. Future research could further improve the robustness of the ISM–BN framework by incorporating larger longitudinal datasets, data-driven structure learning, and dynamic Bayesian networks to better capture temporal variation and reduce reliance on expert-defined relationships.

## 6. Conclusion

The contribution of this study is threefold. First, it provides a systematic, multi-dimensional analysis of the spatial distribution patterns of light‑asset and capital‑intensive traditional retail. Unlike existing research that primarily focuses on a single retail format or relies on linear modeling assumptions, this study integrates objective urban‑environment indicators with human perception factors to capture nonlinear relationships, threshold effects, and interaction mechanisms influencing density of light‑asset and capital‑intensive traditional retail. The proposed framework, combining XGBoost, SHAP, PDP, ISM, and BN, offers a unified approach to uncover hierarchical and probabilistic association pathways across diverse determinants. Second, it reveals fundamental differences in spatial strategies between light‑asset and capital‑intensive traditional retail. While light‑asset traditional retail is characterized by widespread penetration enabled by operational agility and responsiveness to competition and perceptual attributes, capital‑intensive traditional retail adopts selective clustering patterns in high‑yield commercial centers, driven by synergistic facility networks and refined location thresholds. These findings extend understanding of how retail formats position themselves within urban environments, moving beyond descriptive mapping towards an explanatory, mechanism-based account. Third, this study advances methodological practice by linking urban structure with street‑level human perceptions, the analysis bridges a long-standing gap between measurable urban environment and experiential qualities. This dual-perspective framework enables more nuanced interpretations of the socio‑spatial processes shaping retail location decisions.

In short, this research contributes to data‑informed urban retail planning by replacing traditional assumption‑driven models with interpretable machine‑learning techniques capable of modeling complex, context‑dependent relationships. The findings provide urban planners, policy‑makers, and retail strategists with evidence‑based guidance on aligning commercial land‑use policies with retail format characteristics, optimizing accessibility, and balancing competition intensity. Moreover, the framework is adaptable to other sectors and cities, provided that locally relevant variables are incorporated and model parameters are appropriately tuned.

## Supporting information

S1 Table SHAP global variable importance rankings for light-asset retail under 300 m, 500 m, and 800 m grid resolutions. (XLSX)

S2 Table SHAP global variable importance rankings for capital-intensive retail under 300 m, 500 m, and 800 m grid resolutions. (XLSX)

S3 Table Pairwise Spearman’s rank correlation matrix of SHAP importance rankings for light-asset retail. (XLSX)

S4 Table Pairwise Spearman’s rank correlation matrix of SHAP importance rankings for capital-intensive retail. (XLSX)

S5 Table Side-by-side comparison of critical thresholds and 95% bootstrap confidence intervals for core variables across three grid resolutions (light-asset retail). (XLSX)

S6 Table Side-by-side comparison of critical thresholds and 95% bootstrap confidence intervals for core variables across three grid resolutions (capital-intensive retail). (XLSX)

S7 Table Predictive performance of the binary classification submodel in the two-stage hurdle XGBoost. (XLSX)

S8 Table Predictive performance of the truncated regression submodel in the two-stage hurdle XGBoost. (XLSX)

S9 Table Comparison of SHAP variable importance rankings between baseline single-stage XGBoost and hurdle regression stage for light-asset retail top 10 predictors at the 500 m grid. (XLSX)

S10 Table Comparison of SHAP variable importance rankings between baseline single-stage XGBoost and hurdle regression stage for capital-intensive retail top 10 predictors at the 500 m grid. (XLSX)

S11 Table Pairwise Pearson correlation matrix for predictor variables, grouped by accessibility, transit and emotional dimensions. (XLSX)

S12 Table Descriptive statistics. (XLSX)

S13 Table Nonlinear thresholds and interaction intervals identified by PDP analysis. (XLSX)

S1 Data Datasets for light-asset and capital-intensive traditional retail location analysis. (XLSX)

## References

[1] Borgers A, Timmermans H. A decision support and expert system for retail planning. Computers, Environment and Urban Systems. 1991;15(3):179–88. doi: 10.1016/0198-9715(91)90007-z

[2] Kosová R, Lafontaine F. Survival and growth in retail and service industries: evidence from franchised chains. The Journal of Industrial Economics. 2010;58:542–78. doi: 10.1111/j.1467-6451.2010.00431.x

[3] Gao F, Liao S, Jiao Z, Hu Z, Liu Y, Li H, et al. Location differs between traditional and new retail: A comparison analysis of Starbucks and Luckin Coffee in China using machine learning. Cities. 2025;158:105668. doi: 10.1016/j.cities.2024.105668

[4] Dolega L, Lord A. Exploring the geography of retail success and decline: A case study of the Liverpool City Region. Cities. 2020;96:102456. doi: 10.1016/j.cities.2019.102456

[5] Huddleston P, Whipple J, Nye Mattick R, Jung Lee S. Customer satisfaction in food retailing: comparing specialty and conventional grocery stores. International Journal of Retail & Distribution Management. 2009;37(1):63–80. doi: 10.1108/09590550910927162

[6] Reed C, Yu TE, Hughes D. Evaluating the factors influencing the location strategies of specialty grocers versus traditional supermarkets in the United States. Applied Geography. 2023;158:103034. doi: 10.1016/j.apgeog.2023.103034

[7] Bonfrer A, Chintagunta P, Dhar S. Retail store formats, competition and shopper behavior: A Systematic review. Journal of Retailing. 2022;98(1):71–91. doi: 10.1016/j.jretai.2022.02.006

[8] Castillo-Manzano JI, López-Valpuesta L. Urban retail fabric and the metro: A complex relationship. Lessons from middle-sized Spanish cities. Cities. 2009;26(3):141–7. doi: 10.1016/j.cities.2009.02.007

[9] Golovnin OK, Igonina AA. Decision support system for location selection of convenience stores and retail facilities using optimization techniques and GIS. J Phys: Conf Ser. 2021;2134:012015. doi: 10.1088/1742-6596/2134/1/012015

[10] Wood S, Browne S. Convenience store location planning and forecasting – a practical research agenda. Intl J of Retail & Distrib Mgt. 2007;35(4):233–55. doi: 10.1108/09590550710736184

[11] Xu L, Li F, Huang K, Ning J. A two-layer location choice model reveals what’s new in the “new retail”. Annals of the American Association of Geographers. 2023;113:635–57. doi: 10.1080/24694452.2022.2115972

[12] Blennerhassett C, Moore-Cherry N, Bonnin C. Street markets, urban development and immigrant entrepreneurship: Unpacking precarity in Moore Street, Dublin. Urban Studies. 2021;59(13):2739–55. doi: 10.1177/00420980211040928

[13] Che J, Lee JS, Kim S. How has COVID-19 impacted the economic resilience of retail clusters?: Examining the difference between neighborhood-level and district-level retail clusters. Cities. 2023;104457. doi: 10.1016/j.cities.2023.104457 38620167 PMC10300301

[14] Tan Y, Song J, Yu L, Bai Y, Zhang J, Chan MH. The Mechanism of Street Markets Fostering Supportive Communities in Old Urban Districts: A Case Study of Sham Shui Po, Hong Kong. Land. 2024;13:289. doi: 10.3390/land13030289

[15] Davies RL, Rogers DS. Store location and store assessment research. John Wiley & Sons, Ltd. 1984.

[16] Jones KG, Simmons JW. Location, location, location: Analyzing the retail environment. Nelson Canada. 1993.

[17] Wu M, Pei T, Wang W, Guo S, Song C, Chen J, et al. Roles of locational factors in the rise and fall of restaurants: A case study of Beijing with POI data. Cities. 2021;113:103185. doi: 10.1016/j.cities.2021.103185

[18] Gao F, Deng X, Liao S, Liu Y, Li H, Li G, et al. Portraying business district vibrancy with mobile phone data and optimal parameters-based geographical detector model. Sustainable Cities and Society. 2023;96:104635. doi: 10.1016/j.scs.2023.104635

[19] Lamichhane AP, Warren JL, Peterson M, Rummo P, Gordon-Larsen P. Spatial-temporal modeling of neighborhood sociodemographic characteristics and food stores. Am J Epidemiol. 2015;181(2):137–50. doi: 10.1093/aje/kwu250 25515169 PMC4351344

[20] Zhou T, Clapp JM. The location of new anchor stores within metropolitan areas. Regional Science and Urban Economics. 2015;50:87–107. doi: 10.1016/j.regsciurbeco.2014.11.003

[21] Christaller W. Central Places in Southern Germany. Englewood Cliffs, NJ: Prentice-Hall. 1966.

[22] Reilly WJ. The Law of Retail Gravitation. American Journal of Sociology. 1931.

[23] Huff DL. A Probabilistic Analysis of Shopping Center Trade Areas. Land Economics. 1963;39(1):81. doi: 10.2307/3144521

[24] Applebaum W. Guide to store location research: With emphasis on supermarkets. Addison-Wesley Longman. 1968.

[25] Huff DL. Defining and Estimating a Trading Area. Journal of Marketing. 1964;28(3):34–8. doi: 10.1177/002224296402800307

[26] Birkin M, Clarke G, Clarke M. Retail geography & intelligent network planning. John Wiley & Sons. 2002.

[27] Hernández T, Bennison D. The art and science of retail location decisions. International Journal of Retail & Distribution Management. 2000;28(8):357–67. doi: 10.1108/09590550010337391

[28] Currah A. Behind the Web Store: The Organisational and Spatial Evolution of Multichannel Retailing in Toronto. Environ Plan A. 2002;34(8):1411–41. doi: 10.1068/a3562

[29] Weltevreden JWJ, Atzema OALC, Frenken K, de Kruif K, van Oort FG. The Geography of Internet Adoption by Independent Retailers in the Netherlands. Environ Plann B Plann Des. 2008;35(3):443–60. doi: 10.1068/b33032

[30] Kickert C, Vom Hofe R, Haas T, Zhang W, Mahato B. Spatial dynamics of long-term urban retail decline in three transatlantic cities. Cities. 2020;107:102918. doi: 10.1016/j.cities.2020.102918 32921867 PMC7476888

[31] Delage M, Baudet-Michel S, Fol S, Buhnik S, Commenges H, Vallée J. Retail decline in France’s small and medium-sized cities over four decades. Evidences from a multi-level analysis. Cities. 2020;104:102790. doi: 10.1016/j.cities.2020.102790

[32] Ewing R, Handy S, Brownson RC, Clemente O, Winston E. Identifying and Measuring Urban Design Qualities Related to Walkability. J Phys Act Health. 2006;3(s1):S223–40. doi: 10.1123/jpah.3.s1.s223 28834514

[33] Salesses P, Schechtner K, Hidalgo CA. The collaborative image of the city: mapping the inequality of urban perception. PLoS One. 2013;8(7):e68400. doi: 10.1371/journal.pone.0068400 23894301 PMC3722224

[34] Naik N, Philipoom J, Raskar R, Hidalgo C. Streetscore -- Predicting the Perceived Safety of One Million Streetscapes. 2014 IEEE Conference on Computer Vision and Pattern Recognition Workshops. Columbus, OH, USA, 2014. pp. 793–9. 10.1109/CVPRW.2014.121

[35] Dubey A, Naik N, Parikh D, Raskar R, Hidalgo CA. Deep Learning the City: Quantifying Urban Perception at a Global Scale. Lecture Notes in Computer Science. Springer International Publishing. 2016. p. 196–212. doi: 10.1007/978-3-319-46448-0_12

[36] Long Y, Liu L. How green are the streets? An analysis for central areas of Chinese cities using Tencent Street View. PLoS One. 2017;12(2):e0171110. doi: 10.1371/journal.pone.0171110 28196071 PMC5308808

[37] Ye Y, Zeng W, Shen Q, Zhang X, Lu Y. The visual quality of streets: A human-centred continuous measurement based on machine learning algorithms and street view images. Environment and Planning B: Urban Analytics and City Science. 2019;46(8):1439–57. doi: 10.1177/2399808319828734

[38] Harvey C, Aultman-Hall L. Measuring Urban Streetscapes for Livability: A Review of Approaches. The Professional Geographer. 2015;68(1):149–58. doi: 10.1080/00330124.2015.1065546

[39] Lundberg SM, Erion G, Chen H, DeGrave A, Prutkin JM, Nair B, et al. From Local Explanations to Global Understanding with Explainable AI for Trees. Nat Mach Intell. 2020;2(1):56–67. doi: 10.1038/s42256-019-0138-9 32607472 PMC7326367

[40] Rahman NA, Ismail AH, Wong ML, Hamdan FZ. Explainable AI for Urban Retail Site Selection: SHAP-PDP-Bayesian Network Modeling of Facility Synergy and Perceptual Thresholds. JAIAA. 2023;1(3):77–94. doi: 10.63646/jaiaa.2023.010305

[41] Lim F, Rahman NA, Tan HM. Threshold-sensitive business analytics for urban retail density: Interpretable machine learning evidence from multi-source geospatial data. Journal of Business and Data Analytics. 2025;3:90–107. doi: 10.63646/jbda.2025.030205

[42] Yang L, Hou Q, Zhu X, Lu Y, Xu LD. Potential of large language models in blockchain-based supply chain finance. Enterprise Information Systems. 2025;19(11). doi: 10.1080/17517575.2025.2541199

[43] Yang Y, Diez-Roux AV. Walking Distance by Trip Purpose and Population Subgroups. American Journal of Preventive Medicine. 2012;43:11–9. doi: 10.1016/j.amepre.2012.03.015 PMC3377942 22704740

[44] Berry BJL. The Geography of Market Centers and Retail Distribution. Prentice-Hall; 1967.

[45] Berry BJL, Parr JB, Epstein BJ, Ghosh A, Smith RH. Market Centers and Retail Location: Theory and Applications. Prentice-Hall; 1988.

[46] Gao F, Li S, Tan Z, Wu Z, Zhang X, Huang G, et al. Understanding the modifiable areal unit problem in dockless bike sharing usage and exploring the interactive effects of built environment factors. International Journal of Geographical Information Science. 2021;35(9):1905–25. doi: 10.1080/13658816.2020.1863410

[47] Chen T, Guestrin C. XGBoost: A Scalable Tree Boosting System. In: Proceedings of the 22nd ACM SIGKDD International Conference on Knowledge Discovery and Data Mining. San Francisco California USA: ACM; 2016. pp. 785–94. 10.1145/2939672.2939785

[48] Lundberg SM, Lee SI. A unified approach to interpreting model predictions. In: Proceedings of the 31st International Conference on Neural Information Processing Systems, 2017. pp. 4768–77.

[49] Greenwell BM. Pdp: An R package for constructing partial dependence plots. The R Journal. 2017;9:421. doi: 10.32614/rj-2017-016

[50] Thomsen NI, Binning PJ, McKnight US, Tuxen N, Bjerg PL, Troldborg M. A Bayesian belief network approach for assessing uncertainty in conceptual site models at contaminated sites. J Contam Hydrol. 2016;188:12–28. doi: 10.1016/j.jconhyd.2016.02.003 26950254

[51] Formánek T, Sokol O. Location effects: Geo-spatial and socio-demographic determinants of sales dynamics in brick-and-mortar retail stores. Journal of Retailing and Consumer Services. 2022;66:102902. doi: 10.1016/j.jretconser.2021.102902

[52] Seong EY, Lim Y, Choi CG. Why are convenience stores clustered? The reasons behind the clustering of similar shops and the effect of increased competition. Environment and Planning B: Urban Analytics and City Science. 2021;49(3):834–46. doi: 10.1177/23998083211021870

[53] Wu C, Ye Y, Gao F, Ye X. Using street view images to examine the association between human perceptions of locale and urban vitality in Shenzhen, China. Sustainable Cities and Society. 2023;88:104291. doi: 10.1016/j.scs.2022.104291

